# ﻿Geographically structured genetic and morphological variation in a new species of *Cyrtodactylus* (Squamata, Gekkonidae) from a karstic archipelago in western Cambodia

**DOI:** 10.3897/zookeys.1240.139691

**Published:** 2025-06-04

**Authors:** Evan S. H. Quah, L. Lee Grismer, Pablo Sinovas, Phyroum Chourn, Sophea Chhin, Seiha Hun, Anthony Cobos, Peter Geissler, Christian Ching, Matthew L. Murdoch, Sothearen Thi, Jeren J. Gregory, Eddie Nguyen, Alexis P. Hernandez, Amanda Kaatz, Jesse L. Grismer

**Affiliations:** 1 Institute for Tropical Biology and Conservation, Universiti Malaysia Sabah, Jalan UMS, 88400, Kota Kinabalu, Malaysia Universiti Malaysia Sabah Kota Kinabalu Malaysia; 2 School of Biological Sciences, Universiti Sains Malaysia, 11800 Minden, Penang, Malaysia Universiti Sains Malaysia Penang Malaysia; 3 Lee Kong Chian Natural History Museum, National University of Singapore, 2 Conservatory Drive, 117377, Singapore, Singapore National University of Singapore Singapore Singapore; 4 Herpetology Laboratory, Department of Biology, La Sierra University, 4500 Riverwalk Parkway, Riverside, California 92505, USA La Sierra University Riverside United States of America; 5 Department of Herpetology, San Diego Natural History Museum, PO Box 121390, San Diego, California, 92112, USA San Diego Natural History Museum San Diego United States of America; 6 Fauna & Flora Cambodia Programme, 19 Street 360, Phnom Penh, Cambodia Fauna & Flora Cambodia Programme Phnom Penh Cambodia; 7 Department of Biodiversity, General Directorate of Policy and Strategy, Ministry of Environment, Phnom Penh, Cambodia Ministry of Environment Phnom Penh Cambodia; 8 Department of Agronomy, Faculty of Agriculture and Food Processing, ﻿National Meanchey University, Krong Serei Sophaon, Banteay Meanchey Province, Cambodia National Meanchey University Krong Serei Sophaon Cambodia; 9 Department of Evolution, Ecology, and Organismal Biology, University of California, Riverside, CA 92521, USA University of California Riverside United States of America; 10 Zoologisches Forschungsmuseum Alexander Koenig, Adenauerallee 160, D-53113 Bonn, Germany Zoologisches Forschungsmuseum Alexander Koenig Bonn Germany; 11 Museum Natur und Mensch, Gerberau 32, D-79098 Freiburg, Germany Museum Natur und Mensch Freiburg Germany; 12 Department of Life Sciences, Natural History Museum London, Cromwell Road, London SW7 5BD, UK Natural History Museum London London United Kingdom

**Keywords:** Bent-toed gecko, genetics, Indochina, integrative taxonomy, karstic archipelago

## Abstract

A new species of karst-dwelling Bent-toed Gecko (genus *Cyrtodactylus*) is described from an unexplored karstic archipelago in western Cambodia. *Cyrtodactyluskampingpoiensis***sp. nov.** is composed of four allopatric, monophyletic mitochondrial lineages based on the ND2 gene. All are statistically diagnosable from one another based on univariate (ANOVA) and multivariate (PCA, DAPC, and MFA) analyses using a suite of size-corrected morphometric, meristic, and categorical color pattern and morphological characters. Uncorrected pairwise sequence divergence among them is low (1.4–2.2%), indicating a recent divergence from one another. Given their allopatry, diagnosability, monophyly (i.e., no individuals from one population are embedded within another), we contend they are on separate evolutionary trajectories with no chance of secondary overlap via dispersal through the current unhabitual terrain or through the unlikely future coalescence of the karstic formations on which they occur. The discovery of this new species underscores the necessity for further exploration to gain a more informed understanding of the herpetological diversity of Cambodia in general, and that of western Cambodia in particular, where dozens of isolated karstic formations still remain unexplored.

## ﻿Introduction

More than a century and a half ago, the fragmented nature of oceanic archipelagos precipitated some of our earliest notions of allopatric speciation—terms that did not even exist at the time (Darwin 1859; Wallace 1869). These seascapes still continue to enrich our knowledge of evolutionary biology in general (e.g., Williams 1972; Grant and Grant 2002; Losos 2009; Abzhanov 2010) and speciation in particular (e.g., Diamond 1977; Strijk et al. 2012; Reilly et al. 2023). Of late, broader concepts of insular speciation have been widely applied to montane sky-island archipelagos (e.g.,Swann et al. 2005; Bell et al. 2011; Grismer et al. 2015, 2017, 2018a, 2020a, 2023a; He et al. 2019; Quah et al. 2019, [Bibr B68][Bibr B88]) and fragmented habitat islands (e.g., Grismer et al. 2018b, 2021a, 2024a; Kaatz et al. 2021), further enriching our understanding of speciation, its sometimes transitory and murky nature (e.g., Bell et al. 2010; Loredo et al. 2013; Ramírez-Reyes et al. 2020), and the vicissitudes of species concepts (de Queiroz 2007, 2011, 2020; Grismer et al. 2023b; Naomi 2011 and references therein).

**Figure 1.**
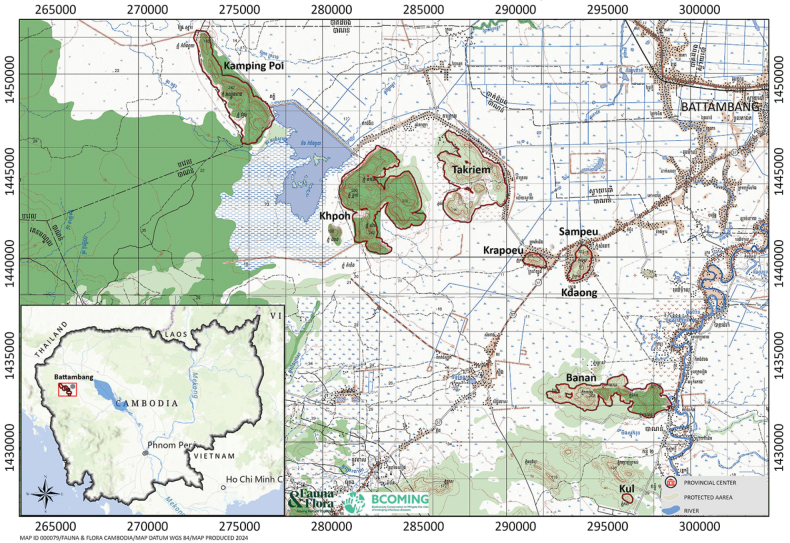
Location of the karstic hills surveyed in Battambang Province and distribution of the new species *Cyrtodactyluskampingpoiensis* sp. nov., from Phnom Banan, and the other populations from Phnom Kamping Poi, Phnom Sampeu, Phnom Khpoh.

The topographical complexity of the Indochinese Peninsula manifests a network of mountain ranges, plateaus, and low-lying basins. Some of the more prominent basins, such as the Ayerwaddy and Salween in Myanmar and the Chao Phraya of Thailand, encompass numerous isolated habitat islands comprised of volcanic, sandstone, and karstic rock, serving as substrates for speciation within the hyperdiverse gekkonid genus *Cyrtodactylus* Gray, 1827 (Grismer et al. 2021b). Some of these basins, such as the Salween in southern Myanmar and the Tonle Sap of western Cambodia, subtend an array of scattered karstic hills, towers, and caves (e.g., Grismer et al. 2018b,c, 2020b, 2021c, 2025), forming vast rocky archipelagos stretching across wide geographic areas. In the Tonle Sap basin and nearby provinces, there are approximately 53 isolated karstic formations whose low-lying landscape is confluent with that of eastern Thailand and its multitude of fragmented karstic formations. During the first herpetological exploration of the Tonle Sap basin’s karstic archipelago in the Banan District of Battambang Province during March of 2024, Grismer et al. (2025) discovered a new species of a karst-dwelling gekkonid of the genus *Hemiphyllodactylus* Bleeker, 1860 that was most closely related to a species from extreme western Thailand. With the new *Hemiphyllodactylus*, four new populations of karst-dwelling *Cyrtodactylus* were also discovered (Fig. 1). Molecular phylogenetic analyses place these populations deep within the *C.intermedius* group (sec. Grismer et al. 2021b) although morphological analyses indicate that they are not conspecific with any of the group’s 15 nominal species (Murdoch et al. 2019; Grismer et al. 2020c, 2021a, 2023c; Chhin et al. 2024; Ampai et al. 2024). Therefore, based on their phylogenetic relationships, morphology, habitat preference, and allopatric distribution, we hypothesize these populations represent one new species that manifest a wide degree of well-structured phylogeographic and morphological variation and is described below.

## ﻿Materials and methods

### ﻿Molecular data

**Figure 2.**
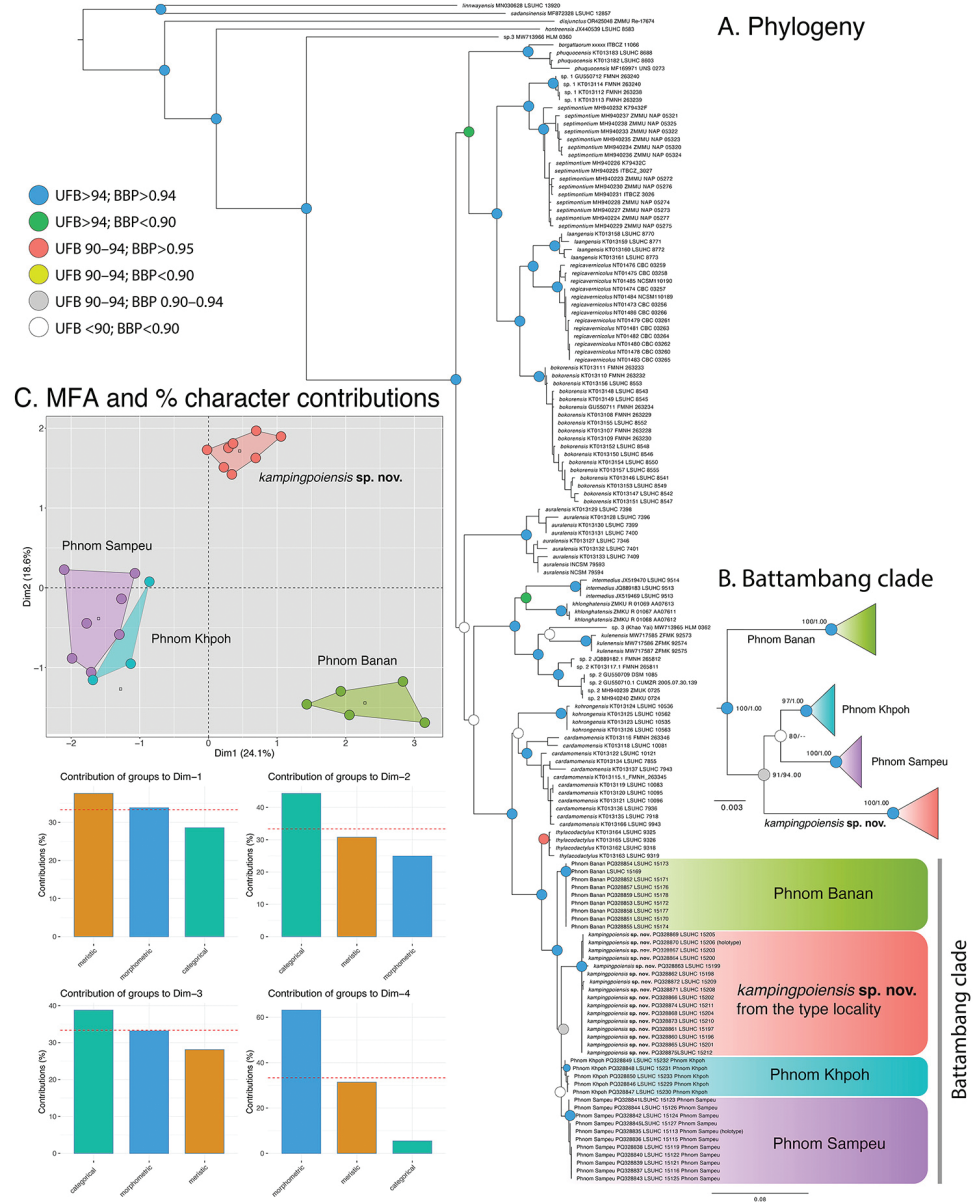
**A** Maximum Likelihood consensus tree of the *Cyrtodactylusintermedius* group **B** enlarged section of the Maximum Likelihood consensus tree highlighting the Battambang clade **C**MFA plots and percent character contribution bar graphs based on the nearly total evidence data set of the Battambang clade.

Liver samples from nine specimens from Phnom Banan, 16 specimens from Phnom Kamping Poi, five specimens from Phnom Khpoh, and 11 specimens from Phnom Sampeu were stored in 95% ethanol. Genomic DNA was isolated using the Qiagen DNeasyTM tissue kit (Valencia, CA, USA). NADH dehydrogenase subunit 2 gene (ND2) and downstream tRNA-Trp, tRNA-Ala, and tRNA-Asn was chosen for phylogeneic analyses with 41 specimens newly sequenced for this work. ND2 was amplified using a double-stranded Polymerase Chain Reaction (PCR) under the following conditions: 2.5 μl genomic DNA (~10–30 ng), 2.5 μl light strand primer (5 μM), 2.5 μl heavy strand primer (5 μM), 1.0 μl dinucleotide pairs (1.0 μM), 2.0 μl 5× buffer (2.0 μM), 1.0 MgCl 10× buffer (1.0 μM), 0.18 μl Taq polymerase (5u/μl), and 9.8s μl ultrapure H2O at n + 1. PCR reactions were executed on a BIO RAD T-100 Thermal Cycler under the following conditions: initial denaturation at 95 °C for 2 min, followed by a second denaturation at 95 °C for 35 s, annealing at 54 °C for 35 s, followed by a cycle extension at 72 °C for 1:35 min repeated for 34 cycles, followed by a final extension cycle run at 68 °C for 7 min. All PCR products were visualized on a 1.0% agarose electrophoresis gel. Successfully targeted PCR products were outsourced to GENEWIZ® for PCR purification, cycle sequencing, and sequencing. Primers used for amplification and sequencing are presented in Murdoch et al. (2019: table 2). Sequences were analyzed from both the 3’ and the 5’ ends separately to confirm congruence between the reads. Both the forward and the reverse sequences were uploaded and edited in GeneiousTM v. 11.1.5 (Drummond et al. 2011) and edited therein. The protein-coding region of the ND2 sequence was aligned by eye. MacClade v. 4.08 (Maddison and Maddison 2015) was used to calculate the correct amino acid reading frame and to confirm the lack of premature stop codons. GenBank accession numbers for all specimens are listed in Fig. 2.

### ﻿Phylogenetic analyses

Maximum likelihood (ML) and Bayesian inference (BI) were used to estimate the phylogenetic relationships of the aligned sequences. The ML phylogeny was estimated using the IQ-TREE webserver (Nguyen et al. 2015; Trifinopoulos et al. 2016) preceded by the selection of substitution models using the Bayesian Information Criterion (BIC) in ModelFinder (Kalyaanamoorthy et al. 2017), which supported HKY+F+G4 as the best fit model of evolution for codon positions 1 and 2, and TPM3u+F+G4 for position 3. Ten-thousand bootstrap pseudoreplicates via the ultrafast bootstrap (UFB; Hoang et al. 2018) approximation algorithm were employed. Nodes having UFB values of 95 and above were considered highly supported (Minh et al. 2013) and nodes with 90–94 UFB values were considered well-supported. The Bayesian inference (BI) analysis was carried out in MrBayes 3.2.3. (Ronquist et al. 2012) on XSEDE using the CIPRES Science Gateway (Cyberinfrastructure for Phylogenetic Research; Miller et al. 2010) employing models of evolution most similar to those above and default priors. Two independent Markov chain Monte Carlo (MCMC) simulations were performed each with four chains, three hot and one cold. We ran the MCMC simulation for 100 million generations, sampled every 10,000 generations, and discarded the first 10% of each run as burn-in. Convergence and stationarity of all parameters from both runs were checked in Tracer v. 1.6 (Rambaut and Drummond 2013) to ensure effective sample sizes (ESS) were above 200. Post-burn-in sampled trees from both runs were combined using the sumt function and a 50% majority rule consensus tree was constructed. Nodes with Bayesian posterior probabilities (BPP) of 0.95 and above were considered highly supported (Huelsenbeck et al. 2001; Wilcox et al. 2002) and nodes BPPs of 0.90–0.94 were considered well-supported. Retaining only ingroup taxa, uncorrected pairwise sequence divergences were calculated in MEGA X (Tamura et al. 2021) using the pairwise deletion option.

### ﻿Species delimitation

The general lineage concept (GLC: de Queiroz 2007) adopted herein proposes that a species constitutes a population of organisms evolving independently from other such populations owing to a general lack of gene flow. By “independently,” it is meant here that new mutations arising in one species cannot readily spread into another species (Barraclough et al. 2003; de Queiroz 2007). Under the GLC, molecular phylogenies were used to recover monophyletic mitochondrial lineages of individual(s) (i.e., populations) in order to develop initial species-level hypotheses – the grouping stage of Hillis (2019). Univariate, multivariate, and discrete color pattern and morphological data were analyzed to search for statistically significant differences in morphology and color patterns that were consistent with the previous species-level hypotheses designations – the construction of boundaries representing the hypothesis-testing step of Hillis (2019). This way, the inherent errors of simultaneously delimiting (phylogeny) and diagnosing (taxonomy) species are avoided (Frost and Hillis 1990; Frost and Kluge 1994; Hillis 2019).

### ﻿Morphological data

#### ﻿Morphometric characters

Measurements were taken on the left side of the body to the nearest 0.1 mm using Mitutoyo dial calipers under a Nikon SMZ 1500 dissecting microscope and follow Grismer and Grismer (2017) and Grismer et al. (2018b). The following measurements were taken: snout-vent length (**SVL**), taken from the tip of the snout to the vent ; tail length (**TL**), taken from the vent to the tip of the tail ; tail width (**TW**), taken at the base of the tail immediately posterior to the postcloacal swelling ; forearm length (**FL**), taken on the ventral surface from the posterior margin of the elbow while flexed 90° to the inflection of the flexed wrist ; tibia length (**TBL**), taken on the ventral surface from the posterior surface of the knee while flexed 90° to the base of the heel ; axilla to groin length (**AG**), taken from the posterior margin of the forelimb at its insertion point on the body to the anterior margin of the hind limb at its insertion point on the body ; head length (**HL**), the distance from the posterior margin of the retroarticular process of the lower jaw to the tip of the snout ; head width (**HW**), measured at the angle of the jaws ; head depth (**HD**), the maximum height of head measured from the occiput to base of the lower jaw ; eye diameter (**ED**), the greatest horizontal diameter of the eye-ball ; eye to ear distance (**EE**), measured from the anterior edge of the ear opening to the posterior edge of the bony orbit ; snout length (**SN**), measured from anteriormost margin of the bony orbit to the tip of snout ; eye to nostril distance (**EN**), measured from the anterior margin of the bony orbit to the posterior margin of the external naris ; interorbital distance (**IO**), measured between the anterior-most edges of the bony orbits ; ear length (**EL**), measured as the greatest vertical distance of the ear opening ; and internarial distance (**IN**), measured between the nares across the rostrum.

#### ﻿Meristic characters

Scale and precloacal pore counts taken were supralabial scales (**SL**) counted from the largest scale immediately below the eyeball to the rostral scale ; infralabial scales (**IL**) counted from the mental to the termination of enlarged scales just after the upturn of the mouth ; the number of paravertebral tubercles (**PVT**) between limb insertions counted in a straight line immediately left or right of the vertebral column ; the number of longitudinal rows of body tubercles (**LRT**) counted transversely across the center of the dorsum from one ventrolateral fold to the other ; the number of longitudinal rows of ventral scales (**VS**) counted transversely across the center of the abdomen from one ventrolateral fold to the other ; the number of expanded subdigital lamellae on the fourth toe (**E4TL**) counted from the base of the first phalanx to the large scale on the digital inflection ; the number of unexpanded subdigital lamellae on the fourth toe (**U4TL**) counted from the digital inflection to the end of the digit and including the claw sheath ; the total number of expanded subdigital lamellae on the fourth toe (**T4TL = E4TL+U4TL**) counted from the base of the first phalanx where it contacts the body of the foot to the claw and including the claw sheath (see Murdoch et al. 2019: fig. 2) ; number of enlarged femoral scales (**FS**) ; total number of enlarged femoral scales from each thigh (**TFS**). In some species, only the distalmost FS are greatly enlarged, and the proximal scales are smaller whereas in others, the enlarged scales are continuous with the enlarged precloacal scales. The separation of the two scales rows was determined to be at a point even with the lateral body margin (see Murdoch et al. 2019: fig. 3). Femoral pores in the new species were absent. The number of enlarged precloacal scales (**PS**) ; the number of precloacal pores in (**PP**) in males (the number of precloacal dimples were recorded in females) ; the number of rows of enlarged post-precloacal scales (**PPS**) on the midline between the enlarged precloacal scales and the granular scales anterior to the vent and the number of postcloacal tubercles (**PCT**). Color pattern meristics taken were the number of dark body bands (**BB**) between the nuchal loop (the dark band running from eye to eye across the nape) and the hind limb insertions ; number of dark-colored (**DCB**) caudal bands ; and the number of light-colored caudal bands (**LCB**).

**Figure 3.**
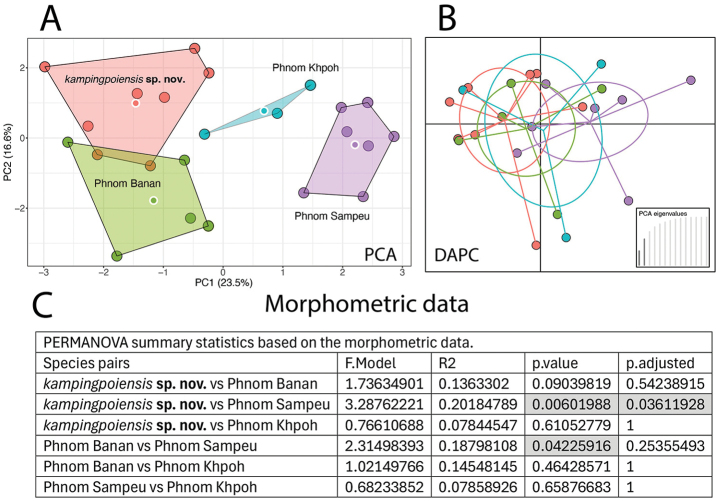
**A, B**PCA and DAPC plots, respectively, of the size-corrected morphometric data of the Battambang clade **C** summary statistics from the PERMANOVA analysis. Shaded cells denote populations whose morphospatial positions are statistically different.

#### ﻿Categorical characters

Categorical morphological and color pattern characters evaluated were body tubercles greatly reduced or not (**Tub-red**) ; body tubercles weakly keeled or raised or moderately to strongly keeled (**Tub-kld**) ; tubercles extending beyond base of tail or not (**CT-ext**) ; subcaudals expanded, being two to three times wider than long or not (**SubC-exp**) ; subcaudals extend onto lateral side of tail or not (**SubC-lat**) ; enlarged femoral and precloacal scales continuous or not (**FS-PS**) ; enlarged proximal femoral scales ~1/2 size of distal femorals or not (**FS-sz**) ; interdigital pocketing present or absent (**Dig-pok**) ; dorsal pattern faded or bold (**DP-fad**) ; distinct dark-colored blotches the on top of the head or not (**HD-mrk**) ; dark dorsal body bands bearing lightened centers or not (**BB-cntr**) ; dark markings in light-colored dorsal interspaces or not (**BB-intr**) ; width of dark-colored body bands thinner or approximately the same width as the light-colored interspaces (**BB-wd**) ; light interspaces reduced to a narrow thin white band or not (**INT-red**) ; dark-colored dorsal bands edged with white tubercles or not (**WHT-tub**) ; dark caudal bands wider than the light caudal bands or not (**DCB-wd**) ; white caudal bands bearing dark markings in adults or not (**WCB-mrk**) and juveniles with or without immaculate white tail tips (**TT-wht**).

### ﻿Statistical analyses

All statistical analyses were conducted using R Core Team (2020). A Levene’s test for meristic and size-corrected morphometric characters was conducted to test for equal variances across all groups. Since all characters had equal variances (*F* ≥ 0.05), they were analyzed by an analysis of variance (ANOVA) and a TukeyHSD *post hoc* test.

A multiple factor analysis (MFA) using the R package *FactorMineR* (Husson et al. 2017) and visualized using the *Factoextra* package (Kassambara and Mundt 2017) was employed for individuals from the four new populations to compare their differences or similarities in morphospace. The MFA was conducted using a concatenated data set comprised of 12 meristic (SL, IL, PVT, LRT, VS, E4TLU4TL, T4TL, TFS, PS, PPS, and PCT), 14 size-corrected morphometric (SVL, FL, TBL, AG, HL, HW, HD, ED, EE, SN, EN, IO, EL, and IN) and four categorical (TT-wht, FS-sz, DP-fad, and BB-wd) characters for a nearly total evidence morphological data set (Suppl. material 1). Not all categorical characters could be used due to their invariability among the four populations. To remove potential effects of allometry in the morphometric characters (see Chan and Grismer 2022), size was corrected using the following equation: X_adj_ = log(X)-β[log(SVL)-log(SVL_mean_)], where X_adj_ = adjusted value; X = measured value; β = unstandardized regression coefficient for each population; and SVL_mean_ = overall average SVL of all populations (Thorpe 1975, 1983; Turan 1999; Lleonart et al. 2000). The morphometrics of each population were size-corrected separately, then concatenated so as not to conflate intra- with interspecific variation (Reist 1986). All data were scaled to their standard deviation to insure they were analyzed based on correlation and not covariance.

MFA is a global, unsupervised, multivariate analysis that incorporates qualitative and quantitative data (Pagès 2015) simultaneously, making it possible to analyze different data types in a nearly total morphological evidence environment. For multispatial diagnostics, MFA is superior to principle component analysis (PCA) in that the data are standardized so the analysis is not biased by a single data type (e.g., morphometric). In an MFA, each individual is described by a different set of variables (i.e., characters) which are structured into different data groups in a global data frame, in this case, quantitative data (i.e., meristic and size-corrected morphometrics) and categorical data (i.e., color pattern and morphological characters). In the first phase of the analysis, separate multivariate analyses are carried out for each set of variables—principal component analyses (PCA) for each quantitative data set and a multiple correspondence analysis (MCA) for the categorical data. The data sets are then normalized separately by dividing all their elements by the square root of their first eigenvalue. For the second phase of the analysis, the normalized data sets are concatenated into a single data frame for a final global PCA. Standardizing the data in this manner prevents one data type from overleveraging another in the second phase. This way, the contributions of each data type to the overall variation in the data set are standardized (Pagès 2015; Kassambara and Mundt 2017).

In order to compare body shapes among the four populations, a PCA and a discriminant analysis of principal components (DAPC) of the 14 size-corrected morphometric characters were employed (Suppl. material 1). PCA is a dimension reducing unsupervised analysis (i.e., all individuals are treated independently) that recovers morphospatial relationships among the sampled individuals (i.e., data points) and how well they form clusters that may or may not align with the putative species boundaries delimited by phylogenetic analyses and diagnosed by the univariate analyses. On the other hand, DAPC from the *adegent* package 2.1.5 in R (Jombart 2021) is a supervised analysis (i.e., groups are specified *a priori* in the analysis) that relies on scaled data calculated from a PCA as a prior step to ensure that variables analyzed are not correlated and number fewer than the sample size. Dimension reduction of the DAPC prior to plotting, was accomplished using PCAtest (Camargo 2022) to recover the principle component (PC) axes which contributed significantly to the overall signal within the PCA. The function runs 1,000 random permutations and bootstrap replicates of the empirical data. Based on the bootstrap resampling and permutation, 95% confidence intervals around mean values are calculated. Significant p-values imply there is non-random correlational structure in the overall dataset and that the PCA is biologically meaningful. Statistically significant PC axes reflect non-random correlations amongst the variables that have a larger contribution beyond random noise. PCA and DAPC analyses were also performed on the meristic data set (Suppl. material 1) as well in order to determine which data type, morphometric or meristic, contributed most to the morphospatial distribution among the four new populations and why from an adaptive standpoint.

A PERMANOVA analysis from the *vegan* package 2.5-3 in R (Oksanen et al. 2020) was used to determine if the centroid locations and group clusters of each species/population from the MFA were statistically different from one another (Skalski et al. 2018) based on the load scores of dimensions 1–5. Using load scores as opposed to raw data, which are normally used, allows for the incorporation of the categorical characters which cannot be run untransformed in a PERMANOVA. All load scores for the PCAs, however, were used. The analysis calculates a Euclidean (dis)similarity matrix using 50,000 permutations. A pairwise *post hoc* test calculates the differences between the populations, generating a Bonferroni-adjusted *p*-value and a pseudo-*F* ratio (*F* statistic). A *p* < 0.05 is considered significant and larger *F* statistics indicate more pronounced group separation. A rejection of the null hypothesis (i.e., centroid positions and the spread of the data points [i.e., clusters] are no different from random) signifies a statistically significant difference between species/populations. This test results in a statistically defensible method of concluding which species/populations plots are actually different from one another and removes the *ad hoc* “eyeballing it” as is normally done.

## ﻿Results

### ﻿Phylogenetic analyses

Both the ML and BI analyses indicate that the four new populations form a strongly supported monophyletic group (UFB 97–100/BPP 1.00), referred to here as the Battambang clade, deeply nested within the *intermedius* group (Fig. 2A, B). The analyses also strongly support (100/1.00) the Battambang clade’s sister lineage relationship to *Cyrtodactylusthylacodactylus*. Additionally, individuals from all four respective populations are strongly recovered as monophyletic (100/1.00; Fig. 2B). Within the Battambang clade, the Phnom (P.) Banan population forms the well-supported sister population (100/1.00) to the remaining three populations collectively. Both analyses recover the P. Kamping Poi population as being the well-supported potential sister population of the P. Sampeu-P. Khpoh lineage (Fig. 2B). The sister population relationship between the P. Sampeu and P. Khpoh populations is poorly supported in the ML analysis and not supported in the BI analysis (80/0.00). Furthermore, the branch length subtending that relationship is so short that these populations, with the P. Kamping Poi population, could be considered as polytomous (Fig. 2A). The Battambang clade has an uncorrected pairwise sequence divergence from the remaining species of the *intermedius* group ranging from 5.0% between the P. Khoh population and *C.cardamomensis* to 24.5% between the P. Sampeu population and *C.disjunctus* (Suppl. material 2). The pairwise sequence divergence among the individuals of the Battambang clade ranges from 1.1–2.2% (Suppl. material 2).

### ﻿Statistical analyses

The PCA of the morphometric data recovered all populations plotting separately in morphospace along the ordination of the first two PCs except for slight overlap between the P. Kamping Poi and P. Banan populations (Fig. 3A). However, the DAPC showed significant overlap in the 66% ellipsoids among all populations (Fig. 3B). Despite their morphospatial separation in the PCA, the PERMANOVA mirrored the DAPC and found significant differences only between the P. Kamping Poi and P. Sampeu populations and between the P. Banan and P. Sampeu populations (Fig. 3C). PC1 accounted for 23.5% of the variation and loaded most heavily for head length (HL) and snout length (SN) and PC2 accounted for 16.6% of the variation and loaded most heavily for eye to ear distance (EE) – all characters relating to dimensions of the head (Table 1).

**Table 1.** Summary statistics of the PCA of the morphometric data. Shaded cells denote heavy loadings. Abbreviations are in the Materials and methods.

	PC1	PC2	PC3	PC4	PC5	PC6	PC7	PC8	PC9	PC10	PC11	PC12	PC13	PC14
Standard deviation	1.81426249	1.52351207	1.36315114	1.22178379	1.04762094	0.97956896	0.93034128	0.79864341	0.78253941	0.56073494	0.53812462	0.37052072	0.30871784	0.16441634
Proportion of Variance	0.23511	0.16579	0.13273	0.10663	0.07839	0.06854	0.06182	0.04556	0.04374	0.02246	0.02068	0.00981	0.00681	0.00193
Cumulative Proportion	0.23511	0.4009	0.53363	0.64026	0.71865	0.78719	0.84901	0.89457	0.93831	0.96077	0.98146	0.99126	0.99807	1
Eigenvalue	3.2915484	2.32108904	1.85818102	1.49275564	1.09750963	0.95955534	0.86553489	0.63783129	0.61236793	0.31442367	0.28957811	0.1372856	0.0953067	0.02703273
SVL	-0.08728	0.0500468	-0.0284522	0.07427331	-0.3499684	0.9248043	-0.0540798	-0.0486213	-0.0183284	0.00795698	-0.0013469	-0.0098121	0.00367259	0.00333359
FL	-0.1531301	-0.3441047	0.14481949	-0.1951079	-0.5764188	-0.1908634	-0.0208145	0.21323317	-0.2275049	0.28047575	-0.4671669	0.09359927	0.15465618	-0.0562567
TBL	-0.3271894	-0.3785061	-0.0445269	-0.2598905	0.02763781	0.03742522	0.11202835	0.22568279	-0.2325245	-0.5588539	0.3132246	0.05395857	0.11845638	0.36478564
AG	-0.1852291	-0.1906489	-0.3016358	0.48438438	0.05154238	0.00130819	0.36353956	0.40412555	0.01269919	0.3808468	0.08355846	-0.2447021	-0.2246075	0.20755435
HL	-0.5066197	0.05677582	-0.0097833	-0.0707779	0.01870923	-0.0299404	-0.2181064	0.18430151	0.17800761	-0.0743037	0.01434615	0.34472187	-0.6055704	-0.3595297
HW	-0.3186456	0.30582029	-0.0406604	-0.3726273	-0.3120084	-0.1440157	0.10092063	-0.1542181	0.04178952	0.11556833	0.31684613	-0.6208108	-0.0337701	-0.1016672
HD	-0.3319735	-0.075737	0.29314555	-0.0243701	0.15490143	0.05259927	0.47675347	-0.4384373	0.30887273	-0.0680971	-0.4259816	-0.0188346	-0.1004616	0.24351563
ED	-0.1816032	0.17394291	-0.1974404	-0.3531401	0.55136477	0.18876592	-0.1663099	0.12631554	-0.4032478	0.2096453	-0.3853305	-0.1724836	0.02863564	0.06490851
EE	-0.1416068	0.48365788	0.04814373	0.31155952	-0.0909937	-0.0687927	0.08796015	0.4036218	0.05068178	-0.4772265	-0.3398241	-0.1554716	0.27435125	-0.1331967
SN	-0.483262	0.13101264	-0.1123776	0.13123944	0.08638236	-0.0437455	-0.0985445	-0.1162697	0.15979576	0.31369436	0.22020116	0.36524096	0.61688598	0.021426
EN	-0.1169942	-0.3589228	0.30215011	0.14873132	0.15124382	0.03500228	-0.5968296	0.09550128	0.36933907	-0.0084095	-0.0465906	-0.4482541	0.10010662	0.07098877
IO	0.05024056	0.41481828	0.51201573	-0.0704161	-0.0742736	-0.018569	-0.1078551	0.26535642	-0.0243783	0.2147458	0.13818274	0.16702613	-0.1702009	0.58966466
EL	-0.2353184	0.04030352	0.10864139	0.49214159	-0.0839475	-0.1408888	-0.2384656	-0.4361197	-0.6090493	-0.0941023	0.01336535	-0.0731026	-0.154845	0.07573385
IN	-0.0316986	-0.1145348	0.61512654	0.03881673	0.2548841	0.13712725	0.31156131	0.16068648	-0.2572595	0.12349781	0.24639739	-0.0475584	0.11847151	-0.4908014

**Figure 4.**
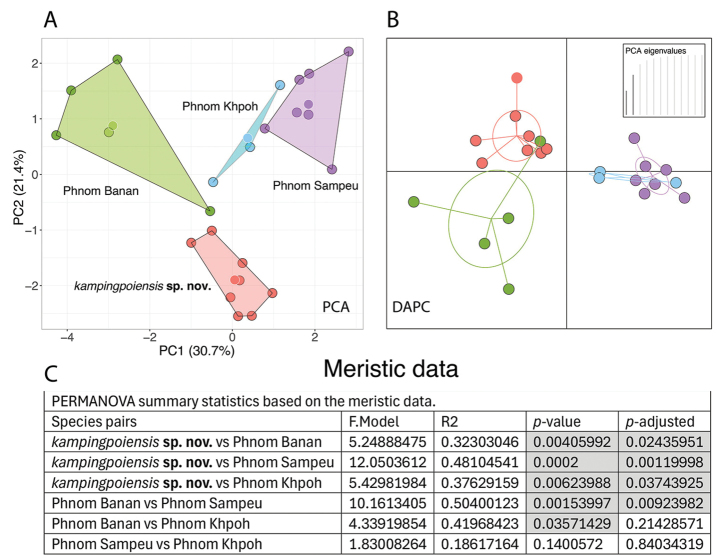
**A, B**PCA and DAPC plots, respectively, of the meristic data of the Battambang clade **C** summary statistics from the PERMANOVA analysis. Shaded cells denote populations whose morphospatial positions are statistically different.

Conversely, the PCA of the meristic data recovered nearly all populations plotting completely separately in morphospace along the ordination of the first two PCs which was mirrored in the DAPC (Fig. 4A, B). Corroborating these differences, the PERMANOVA analysis recovered all populations as being significantly different from one another except for P. Sampeu and P. Khpoh. Most were significantly different at the more discerning *p*-adjusted value (Fig. 4C). PC1 accounted for 30.7% of the variation and loaded most heavily for the total number of 4^th^ toe lamellae (T4TL), number of expanded 4^th^ toe lamellae (E4TL), and the number of paravertebral tubercles (PVT). PC2 accounted for 21.4% of the variation and loaded most heavily for the total number of enlarged precloacal scales (PS), number of longitudinal rows of tubercles (LRT), number of unexpanded 4^th^ toe lamellae (U4TL), and number of ventral scales (VS) (Table 2).

**Table 2.** Summary statistics of the PCA of the meristic data. Shaded cells denote heavy loadings. Abbreviations are in the Materials and methods.

	PC1	PC2	PC3	PC4	PC5	PC6	PC7	PC8	PC9	PC10	PC11
Standard deviation	1.91874971	1.59763806	1.1898923	1.06797909	1.02352442	0.88121977	0.76632845	0.55031044	0.51142314	0.36292899	0.3111846
Proportion of Variance	0.3068	0.2127	0.11799	0.09505	0.0873	0.06471	0.04894	0.02524	0.0218	0.01098	0.00807
Cumulative Proportion	0.3068	0.5195	0.63749	0.73254	0.81984	0.88455	0.93349	0.95873	0.98052	0.9915	0.99957
eigen	3.68160045	2.55244737	1.41584369	1.14057934	1.04760225	0.77654829	0.58725929	0.30284158	0.26155363	0.13171745	0.09683585
SL	0.17326323	0.1651552	-0.5603096	0.46263365	0.14062141	0.03707266	-0.2448837	0.0697198	-0.489553	-0.2080258	0.21266337
IL	-0.0254806	0.30734234	0.11506108	-0.223321	0.60538921	-0.5861433	0.0353749	-0.2511456	-0.1424952	0.11792664	0.18069266
PVT	-0.3574612	-0.1590297	-0.4316934	-0.2373959	0.08116444	-0.1272866	-0.34542	-0.0933	-0.0699248	0.10195704	-0.6587795
LRT	-0.304204	-0.4022792	-0.0783882	-0.1025981	-0.0191965	-0.2049428	-0.4457324	0.32772103	0.19349745	0.12786903	0.56922023
VS	-0.2765739	-0.3501424	0.3034379	0.14978253	-0.1011264	-0.3799279	0.24118126	0.20153712	-0.4296907	-0.4770117	-0.1426858
E4TL	-0.3880702	-0.0232155	-0.2225963	0.33878332	-0.2689796	-0.1628965	0.39521929	-0.3118024	-0.0165758	0.40662509	0.17585339
U4TL	-0.3284321	0.34089938	-0.0990162	-0.4231148	0.00715937	0.2565798	0.06691444	0.13125078	-0.0407721	-0.4208345	0.20173025
T4TL	-0.4411792	0.2264118	-0.2074036	-0.1308085	-0.0852898	0.15084066	0.26348799	-0.0613201	-0.0008857	-0.0755263	0.11326445
TFS	0.13682386	-0.4493157	0.00078358	-0.4134166	0.072499	0.34906154	0.12200423	-0.2563252	-0.5589212	0.22837241	0.19000569
PS	-0.2887671	0.32611163	0.38903119	0.15090559	0.06187766	0.2272462	-0.1784263	0.4130475	-0.3672148	0.47879216	-0.1184337
PPS	-0.1068566	-0.2886927	-0.1519137	0.19829433	0.67616041	0.259687	0.39643927	0.30753507	0.23745782	0.02642696	-0.0780445
PCT	-0.3254046	-0.0946321	0.33382986	0.32072144	0.22492959	0.31059532	-0.3586545	-0.5711791	0.08662861	-0.2414301	0.05563348

The MFA results of the near total evidence morphological data set was very similar to the PCA and DAPC of the meristic data in that all populations plotted separately along the first two dimensions (Fig. 2C). It would be tempting to say that this was a result of the meristic data, however those data contributed to only ~37% of the 24.1% of the variation in dimension1 (Dim 1) (Fig. 2C). The categorical data contributed to the majority of variation in Dim 2 and Dim 3 and the morphometric data contributed the majority of variation in Dim 4. The PERMANOVA analysis also recovered all populations as significantly different from one another at the unadjusted *p*-value, except for except for the P. Sampeu and P. Khpoh populations, and most were significantly different at the *p*-adjusted value (Fig. 4C).

Results of the ANOVA analysis recovered significant differences in both meristic and morphometric characters among the four populations in a varying number of population comparisons across a varying number of characters (Table 3).

**Table 3.** Statistically significant differences (p < 0.05) in meristic and morphometric character means for all combinations of population pairs based on ANOVA analyses and subsequent TukeyHSD tests. Blank cells lacking p-values indicate no significant difference. P. = Phnom. Character abbreviations are in the Materials and methods.

	Phnom Banan	*C.kampingpoiensis* sp. nov.	Phnom Sampeu	Phnom Khpoh		Phnom Banan	*C.kampingpoiensis* sp. nov.	Phnom Sampeu	Phnom Khpoh
**meristic data**					**morphometric data**				
**LRT**					**EE**				
Phnom Banan					Phnom Banan		0.01852		
*C.kampingpoiensis* sp. nov.				0.00017	*C.kampingpoiensis* sp. nov.				
Phnom Sampeu	1.97E-05	8.09E-08			Phnom Sampeu		0.01616		
Phnom Khpoh	0.01051				Phnom Khpoh				
**PCT**					**HL**				
Phnom Banan					Phnom Banan				
*C.kampingpoiensis* sp. nov.					*C.kampingpoiensis* sp. nov.				
Phnom Sampeu	0.00755				Phnom Sampeu	0.00054	0.00001		
Phnom Khpoh					Phnom Khpoh		0.01229		
**PS**					**HW**				
Phnom Banan		0.00137			Phnom Banan				
*C.kampingpoiensis* sp. nov.					*C.kampingpoiensis* sp. nov.				
Phnom Sampeu	0.00137				Phnom Sampeu		0.01212		
Phnom Khpoh					Phnom Khpoh				
**PVT**					**SN**				
Phnom Banan					Phnom Banan		0.00330		
*C.kampingpoiensis* **sp. nov.**					*C.kampingpoiensis* **sp. nov.**				
Phnom Sampeu	0.00038	0.00883			Phnom Sampeu		0.00006		
Phnom Khpoh	0.00050	0.00734			Phnom Khpoh		0.00621		
**TFS**					**TBL**				
Phnom Banan					Phnom Banan		0.02995		
*C.kampingpoiensis* sp. nov.					*C.kampingpoiensis* sp. nov.				
Phnom Sampeu					Phnom Sampeu		0.00080		
Phnom Khpoh	0.00117	0.00000			Phnom Khpoh		0.02534		
**E4TL**									
Phnom Banan									
*C.kampingpoiensis* **sp. nov.**									
Phnom Sampeu	0.00859								
Phnom Khpoh									
Phnom Banan		0.00030							
*C.kampingpoiensis* **sp. nov.**									
Phnom Sampeu									
Phnom Khpoh	0.01097								
**T4TL**									
Phnom Banan									
*C.kampingpoiensis* **sp. nov.**		0.00007		0.02232					
Phnom Sampeu									
Phnom Khpoh	0.00014								
**VS**									
Phnom Banan									
*C.kampingpoiensis* **sp. nov.**									
Phnom Sampeu									
Phnom Khpoh	0.04279	0.00768							
Phnom Banan									

### ﻿Taxonomy

All the newly discovered populations are monophyletic and none are phylogenetically nested within any other species of the *intermedius* group nor are they sister to any of the group’s nominal species (Fig. 2A). With the exception of the P. Sampeu and P. Khpoh populations, they all bear statistically significant morphospatial differences from one another in the MFA and the PCAs (Figs 2–4) and have significantly different mean values from one another in varying numbers of morphometric and meristic characters (Table 3) as well as differing categorically (Suppl. material 3). The polytomous relationship among the P. Kamping Poi, P. Sampeu, and P. Khpoh populations and the absence of characters separating the latter two that differ only by a pairwise sequence divergence of only 1.1%, indicates they all could be considered the same species despite their allopatry and with no chance of dispersal (see discussion). The short branch lengths subtending the inter-nodes of the other populations in the Battambang clade may indicate the same. Therefore, at this point, all populations are considered conspecific and described as a single species. The type series is designated from the P. Kamping Poi population due to its larger sample size, while the remaining three populations are treated as geographic variants.

#### ﻿ Cyrtodactylus kampingpoiensis sp. nov.

Taxon classificationAnimaliaSquamataGekkonidae

B6C6C44A-C6ED-5C0E-B5A2-A31253986196

https://zoobank.org/F5827770-1118-4C6D-8930-F3DC4E8FDD42

Figs 5 , 6 , 7

##### Type material.

***Holotype*** • Adult male (LSUHC 15206) collected from Phnom Kamping Poi, Banan District, Battambang Province, Cambodia at 13°5.795'N, 102°55.798'E, at 114 m and nearby areas on 22 March 2024 by Pablo Sinovas, Seiha Hun, Phyroum Chourn, Matthew L. Murdoch, L. Lee Grismer, Evan S. H. Quah, Sothearen Thi, Christian Ching, and Anthony Cobos. ***Paratypes*** • Two adult males (LSUHC 15203 and 15211) and five adult females (LSUHC 15205, 15207, 15209–10, and 15212) bear the same collection data as the holotype. The type series was collected from 1530–2200 hrs.

**Figure 5.**
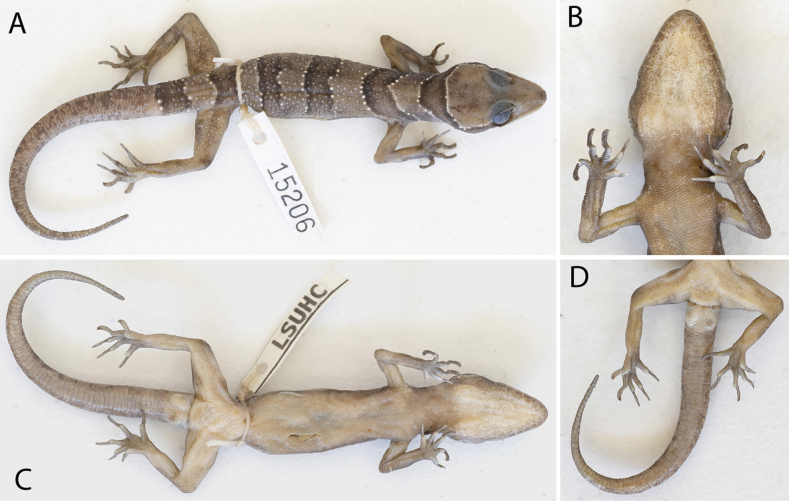
Adult male holotype of *Cyrtodactyluskampingpoiensis* sp. nov. LSUHC 15206 **A** dorsal view **B** ventral view of gular region, throat, forelimbs, and feet **C** ventral view **D** ventral view of tail, pelvic region, hind limbs, and feet.

##### Additional specimens examined.

• Six hatchlings (LSUHC 15196– 200, and 151202) bear the same collection data as the type series. The specimens were too small to recover reliable morphometric and meristic data but were included in the phylogenetic analyses.

**Figure 6.**
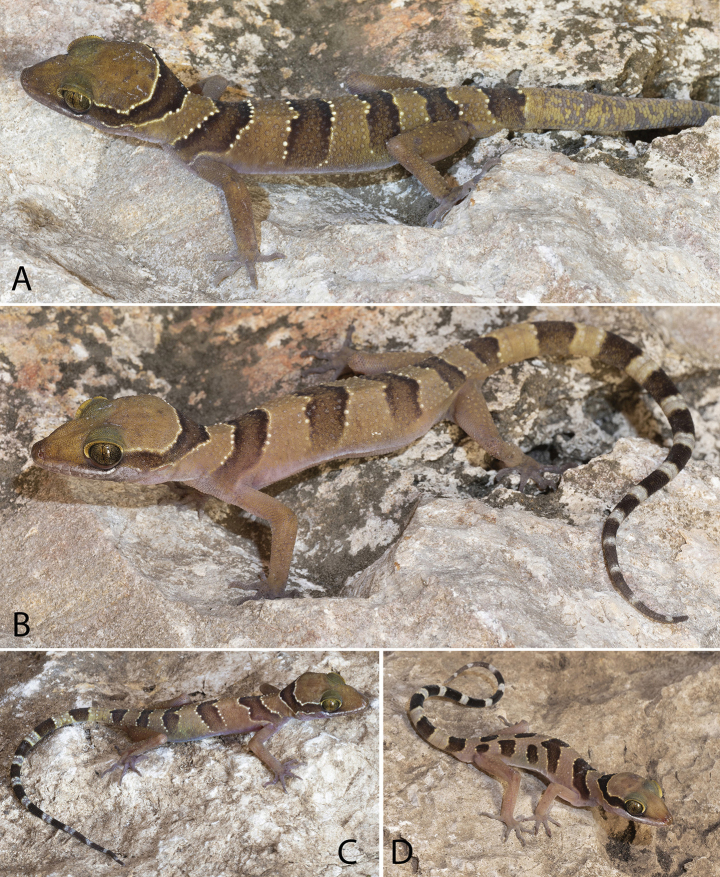
*Cyrtodactyluskampingpoiensis* sp. nov. **A** adult male holotype LSUHC 15206 **B** gravid adult female LSUHC 15207 **C** adult female LSUHC 15205 **D** juvenile LSUHC 15176 **E** juvenile male LSUHC 15203.

##### Diagnosis.

**Figure 7.**
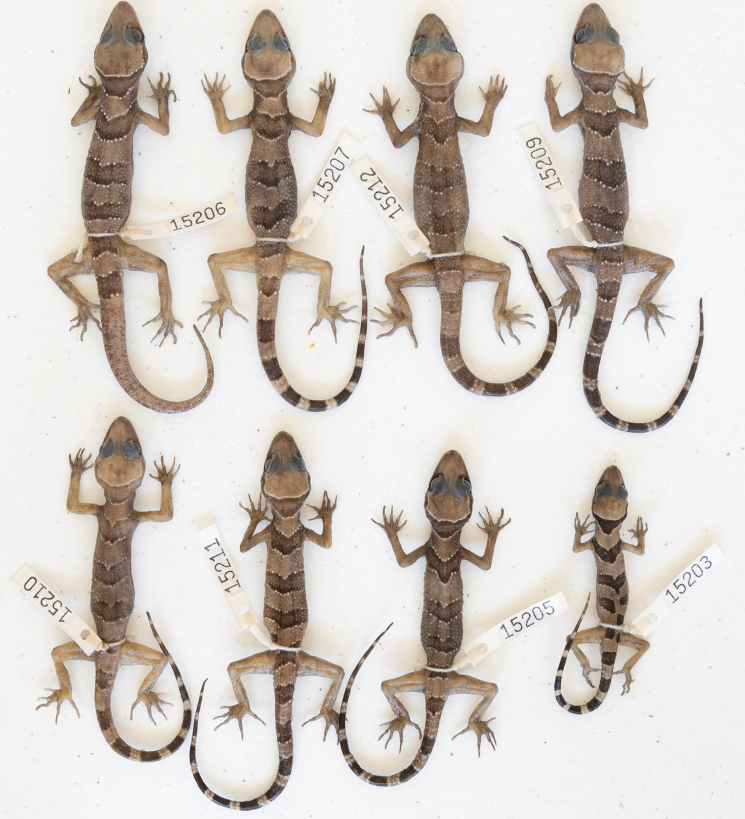
Type series of *Cyrtodactyluskampingpoiensis* sp. nov.

*Cyrtodactyluskampingpoiensis* sp. nov. can be separated from all other species of the *intermedius* group by the combination of having a maximum SVL of 79.6 mm (female); 9–11 supralabials; nine or 10 infralabials; 30–37 paravertebral tubercles; 19–21 rows of longitudinally arranged tubercles; 38–46 longitudinal rows of ventrals; 5–7 expanded subdigital lamellae on the fourth toe; 11–13 unmodified subdigital lamellae on the fourth toe; 18–20 total subdigital lamellae on the fourth toe; 26–34 total number of enlarged femorals; no femoral pores; 5–9 enlarged precloacals; 7–9 precloacal pores in males (*n* = 3); three or four rows of large post-precloacal scales; 0–3 postcloacal tubercles; four dark body bands; 11–13 dark caudal bands (*n* = 7); 10–12 light caudal bands (*n* = 7); body tubercles not greatly reduced and moderately keeled; caudal tubercles extend beyond base of tail; subcaudals transversely expanded but not extending up onto side of tail; enlarged femorals and enlarged precloacals not continuous; enlarged proximal femorals equal (rarely subequal, one of seven) in size to distal femorals; no interdigital pockets; dorsal pattern not faded; no distinct dark markings on the top of head; lightened centers in dark body bands variable; no dark markings in light interspaces between body bands; dark body bands equal in width to light interspaces; light interspaces not reduced to a narrow thin white band; dark body bands bordered by prominent white tubercles; dark caudal bands slightly wider than light caudal bands; light caudal bands bearing dark markings in adults; posterior margin of nuchal loop not smoothly rounded (Table 4; Fig. 7; Suppl. materials 3, 4).

**Table 4.** Data for the type series of *Cyrtodactyluskampingpoiensis* sp. nov. from Phnom Kamping Poi, Battambang Province, Cambodia.

Specimen	LSUHC 15206 holotype	LSUHC 15210 paratype	LSUHC 15205 paratype	LSUHC 15209 paratype	LSUHC 15207 paratype	LSUHC 15211 paratype	LSUHC 15203 paratype	LSUHC 15212 paratype
sex	m	f	f	f	f	m	m	f
**Meristic characters**								
supralabials (SL)	9	11	10	11	11	10	10	11
infralabials (IL)	9	9	10	9	9	10	9	10
paravertebral tubercles (PVT)	32	37	34	34	35	31	30	34
longitudinal rows of tubercles (LRT)	20	21	20	19	20	20	20	21
longitudinal rows of ventral scales (VS)	43	41	42	41	38	42	46	44
expanded subdigital lamellae on 4^th^ toe (T4LE)	5	7	6	7	7	7	7	7
unmodified subdigital lamellae on 4^th^ toe (T4LU)	13	13	12	11	12	12	11	12
total subdigital lamellae on 4^th^ toe (T4TL)	18	20	18	19	19	19	18	19
enlarged femoral scales (R,L)	16+18	15+14	17+13	14+15	13+15	13+15	13+13	13+14
total enlarged femoral scales (TFS)	34	29	30	29	28	28	26	27
femoral pores	0	0	0	0	0	0	0	0
enlarged precolacal scales (PS)	9	5	6	7	6	9	8	9
precloacal pores (PP)	3L, 4R, gap 2	0	0	0	0	9	8	0
post-precloacal scale rows (PPS)	3	3	3	4	3	3	3	3
postcloacal tubercles (PCT)	3	0	2 small	3 small	3 small	3	3	3
**Categorical characters**								
subcaudals expanded	yes	yes	yes	yes	yes	yes	yes	yes
subccaudals extend up onto lateral side of tail	no	no	no	no	no	no	no	no
body dark bands (BB)	4	4	4	4	4	4	4 broken	4
dark caudal bands (DCB)	/	11	12	12	13	11	11	12
light caudal bands (LCB)	/	10	11	11	12	10	10	11
body tubercles greatly reduced (Tub-red)	no	no	no	no	no	no	no	no
body tubercles moderately keeled (Tub-kld)	yes	yes	yes	yes	yes	yes	yes	yes
caudal tubercles extend beyond base of tail (CT-ext)	yes	yes	yes	yes	yes	yes	yes	yes
subcaudals expanded (SubC-exp)	yes	yes	yes	yes	yes	yes	yes	yes
subccaudals extend up onto lateral side of tail (SubC-lat)	no	no	no	no	no	no	no	no
enlarged femoral and precloacal scales continuous (FS-PS)	no	no	no	no	no	no	no	no
enlarged proximal femoral < 1/2 size of distal femorals (FS-sz)	equal	equal	equal	equal	equal	equal	equal	subequal
pocketing between digits of hind feet (Dig-pok)	no	no	no	no	no	no	no	no
dorsal pattern faded (CP_fd)	no	no	no	no	no	no	no	no
distinct dark pigmented blotches on top of head present (HD-mrk)	no	no	no	no	no	no	no	no
dark body bands with lightened centers (BB-cntr)	yes	weak	weak	weak	weak	weak	no	no
dark body markings in light interspaces (BB-intr)	no	no	no	no	no	no	no	no
dark dorsal bands thin or ~ same width as light interspaces (BB-wd)	equal	equal	equal	equal	equal	equal	equal	equal
light interspaces reduced to a narrow thin white band	no	no	no	no	no	no	no	no
dark dorsal bands bordered by prominently colored white tubercles (Wht-tub)	yes	yes	yes	yes	yes	yes	yes	yes
dark caudal bands wider than light caudal bands (DCB-wd)	yes	yes	yes	yes	yes	yes	yes	yes
light caudal bands bearing dark markings in adults (WCB-mrk)	yes	yes	yes	yes	yes	yes	yes	yes
**Morphometric characters (mm)**								
SVL	78.9	71.1	71.4	79.6	77.9	71.0	53.0	79.0
TaiL	82.0	90.0	92.0	103.0	105.0	95.0	68.0	no tail
TaiW	7.3	6.6	5.8	7.4	6.8	5.8	4.4	6.4
FL	12.2	11.9	11.2	12.4	12.8	10.7	8.2	12.6
TBL	15	14.1	13.9	15.7	14.6	13.8	9.6	14.8
AG	35.6	34.5	32.8	36.6	34.9	32.6	23.4	36.0
HL	22.5	20.3	20.6	22.8	22.2	20.1	16.4	22.6
HW	15.0	13.9	13.4	15.6	15.3	14.3	10.3	16.3
HD	8.7	9.0	8.4	10.2	8.4	7.8	6.4	9.9
ED	5.1	4.5	4.6	4.9	4.9	4.8	3.7	5.2
EE	6.8	5.8	5.5	6.1	6.2	5.8	4.6	6.3
SN	8.5	7.9	8.0	8.8	8.6	8.0	6.3	9.0
EN	6.2	5.6	5.9	6.3	6.4	4.8	4.4	6.3
IO	5.1	4.8	4.4	5.1	5.4	4.2	3.9	5.2
EL	1.9	2.1	2.1	2.2	2.3	1.7	1.6	1.9
IN	2.2	2.5	2.3	2.4	2.3	1.9	1.7	2.3

##### Description of holotype.

(Fig. 5; Table 4). Adult male SVL 78.9 mm; head moderate in length (HL/SVL 0.29), width (HW/HL 0.67), somewhat flattened (HD/HL 0.39), distinct from neck, triangular in dorsal profile; lores weakly concave anteriorly, weakly inflated posteriorly; prefrontal concave; canthus rostralis rounded; snout elongate (SN/HL 0.38), slightly convex, rounded in dorsal profile; eye large (ED/HL 0.23); ear opening elliptical, obliquely oriented, moderate in size; eye to ear distance slightly greater than diameter of eye; rostral rectangular, partially divided, bordered posteriorly by large left and right supranasals and a small internasal, bordered laterally by first supralabials; external nares bordered anteriorly by rostral, dorsally by large supranasal, posteriorly by two moderately sized postnasals, bordered ventrally by first supralabial; nine (R,L) rectangular supralabials tapering abruptly to below posterior margin of eye, first–sixth supralabials largest; nine (R, L) infralabials tapering smoothly to slightly past the termination of enlarged supralabials to corner of mouth; scales of rostrum and lores flat, much larger than granular scales on top of head and occiput; scales of occiput intermixed with small, rounded, tubercles; superciliaries elongate, largest dorsally; mental triangular, bordered laterally by first infralabials and posteriorly by large left and right trapezoidal postmentals contacting medially for ~ 40% of their length posterior to mental; one row of enlarged, sublabials extending posteriorly to seventh infralabials (R, L); gular and throat scales small, granular, grading posteriorly into slightly larger, flatter, smooth, imbricate, pectoral and ventral scales.

Body relatively long (AG/SVL 0.45) with well-defined ventrolateral folds; dorsal scales small, granular, interspersed with moderately sized, keeled, semi-regularly arranged tubercles extending from occiput to beyond base of tail; ~ 20 longitudinal rows of tubercles at midbody; ~ 32 paravertebral tubercles; 43 flat, imbricate, ventral scales much larger than dorsal scales; seven enlarged pore-bearing precloacal scales, 4R, 3L separated by two poreless scales; no deep precloacal groove or depression; and three rows of large post-precloacal scales on midline.

Forelimbs moderate in length and stature (FL/SVL 0.15); granular scales of forelimbs larger than those on body, small rounded tubercles on dorsal surface of forearms; palmar scales rounded, juxtaposed; digits well-developed, inflected at basal interphalangeal joints, slightly narrower distal to inflections; subdigital lamellae transversely expanded, those proximal to joint inflections much wider than the nearly unmodified lamellae distal to inflections; claws well-developed, sheathed by a dorsal and ventral scale; hind limbs robust, wider and longer than forelimbs (TBL/SVL 0.19), covered dorsally by granular scales interspersed with moderately sized tubercles, larger and flatter scales anteriorly; ventral scales of thighs flat, imbricate, larger than dorsals; subtibial scales, flat, imbricate, slightly smaller than those of thigh; one row of 16(R)18(L) slightly enlarged femoral scales terminating distally before knee, not continuous with enlarged precloacal scales, and poreless; proximal femorals nearly same size as distal femorals, all femorals forming an abrupt union with smaller, granular, posteroventral scales of thigh; plantar scales flat, juxtaposed; digits well-developed, inflected at basal interphalangeal joints; claws well-developed, sheathed by a dorsal and ventral scale at base; and five (R,L) wide subdigital lamellae on fourth toe proximal to joint inflection, 13 (R,L) narrower lamellae distal to joint inflection, 18 total subdigital lamellae.

Tail long (TL/SVL 1.04), partially regenerated, original portion 9.6 mm, regenerated portion 72.4 mm, 7.3 mm wide at base, tapering to a point; dorsal caudal scales of original portion of tail small, generally square, juxtaposed; median row of subcaudals significantly larger than dorsal caudals, transversely expanded, not extending dorsally onto lateral side of tail; body tubercles extending slightly beyond base of tail; hemipenal swellings at base of tail, three large postcloacal tubercles on both sides; and postcloacal scales flat, imbricate.

##### Color and pattern.

(Figs 5–7). Ground color of top of head, limbs, and dorsum straw to light-brown; top of head immaculate; prominent dark-brown nuchal loop edged in light-yellow bearing a somewhat flat posterior border extends between postorbital regions across the nape; no dark-brown band on nape; four immaculate dark-brown, slightly wavy-edged, dorsal body bands edged with bright-white tubercles have slightly lightened centers and are equal in width to straw-colored interspaces, extend from shoulders to groin, terminating ventrally slightly above the ventrolateral folds; first dorsal band extends anteriorly across shoulders; light-colored dorsal interspaces immaculate; forelimbs and hind limbs generally immaculate; one dark-brown caudal band on original portion of tail; regenerated portion of tail straw-colored overlain with faint, dark, irregularly shaped markings; iris gold with thin black reticulations; venter beige with faint, dark shadowing on lateral edges of belly, limbs, and throat; and subcaudal region essentially unicolor light-brown.

##### Etymology.

The species name *kampingpoiensis* is in reference to the type locality at Phnom Kamping Poi, Banan District, Battambang Province, Cambodia (Fig. 1).

##### Distribution.

The type series of *Cyrtodactyluskampingpoiensis* sp. nov. is known only from the type locality at Phnom Kamping Poi, Banan District, Battambang Province, Cambodia (Fig. 1).

##### Variation.

(Figs 6, 7). The paratypes remarkedly approach the holotype in general coloration and pattern. The most notable variation pertains to the dorsal banding of the juvenile LSUHC 15203 whose third and fourth bands are broken on the midline and offset (Fig. 6). The overall pattern of LSUHC 15212 is far less bold than that of the holotype and the medial portion of its first band is posteriorly protracted. The juveniles have darker bands lacking lightened centers and are edged with yellow tubercles and there are no dark markings in the caudal interspaces. This is all accentuated in the hatchlings whose coloration is often slightly faded in the posterior ~ 25% of the tail. The precloacal pore series in the holotype is separated medially by two poreless scales whereas the pore-bearing precloacal scales on the paratypes are continuous. Differences in meristics, morphometrics, and categorical characters are detailed in Table 4 and Suppl. material 3.

##### Geographic variation.

(Tables 3, 5). Potential diagnostic differences in maximum SVL, meristics, and categorical characters between *Cyrtodactyluskampingpoiensis* sp. nov. and all other species in the *intermedius* group – except for species in the Battambang clade (used here in reference to all four populations) – are detailed in Suppl. material 4. *Cyrtodactyluskampingpoiensis* sp. nov. has an uncorrected pairwise sequence divergence from the *intermedius* group species ranging from 3.5–23.6% (Suppl. material 2). *Cyrtodactyluskampingpoiensis* sp. nov. bears a 1.1–2.2% uncorrected pairwise sequence divergence from the other three populations. In meristics, it differs further from the P. Banan population in having a statistically significant fewer number of enlarged precloacals (PS; 5–9 vs 9–12), unmodified subdigital lamellae (U4TL; 11–13 vs 13–16) and total number of subdigital lamellae (T4TL; 18–20 vs 20–23) (Table 5). It differs further in morphometrics in having a statistically significant longer ear-eye distances (EE), longer snout (SN), and a shorter foreleg (TBL). The PERMANOVA analyses indicate that *Cyrtodactyluskampingpoiensis* sp. nov. differs significantly in morphospace from the P. Banan population in the MFA (Figs 2B, 4C) and the meristic PCA (Fig. 4C). Categorically, *C.kampingpoiensis* sp. nov. differs from the P. Banan population in that its slightly enlarged femorals and enlarged precloacals are usually discontinuous versus being continuous (four of five specimens; Suppl. materials 3, 4); the dark body bands are equal in width to the light interspaces as opposed to being wider; and hatchings and small juveniles have only a slightly faded tail tip as opposed to a nearly immaculate white tail tip (Fig. 6D vs Fig. 9E).

**Table 5.** Summary statistics for *C.kampingpoiensis* sp. nov., the Phnom Banan population, the Phnom Sampeu population, and the Phnom Khpoh population.

Phnom Banan														
**meristics**	**SL**	**IL**	**PVT**	**LRT**	**VS**	**T4LE**	**T4LU**	**T4TL**	**TFS**	**PS**	**PPS**	**PCT**		
mean	10.2	9.8	35.4	19.2	41.6	7.4	14.4	21.8	24.8	10.2	2.8	3.2		
sd	0.84	0.84	2.30	0.84	2.41	0.55	1.14	1.30	4.09	1.10	0.45	0.45		
min	9	9	33	18	39	7	13	20	19	9	2	3		
max	11	11	38	20	45	8	16	23	30	12	3	4		
N	5	5	5	5	5	5	5	5	5	5	5	5		
**morphometrics**	**SVL**	**FL**	**TBL**	**AG**	**HL**	**HW**	**HD**	**ED**	**EE**	**SN**	**EN**	**IO**	**EL**	**IN**
mean	1.8477	1.0691	1.1673	1.5267	1.3165	1.1360	0.9285	0.6818	0.7279	0.9067	0.7826	0.6307	0.2856	0.3507
sd	0.05514	0.02585	0.01116	0.03584	0.00607	0.01535	0.01345	0.00718	0.03343	0.00724	0.00917	0.04445	0.05414	0.01534
min	1.7536	1.0324	1.1505	1.4994	1.3092	1.1126	0.9133	0.6724	0.6718	0.8985	0.7768	0.5906	0.1998	0.3344
max	1.8971	1.0999	1.1793	1.5822	1.3255	1.1484	0.9488	0.6897	0.7579	0.9184	0.7986	0.6915	0.3422	0.3713
N	5	5	5	5	5	5	5	5	5	5	5	5	5	5
*Cyrtodactyluskampingpoiensis* sp. nov														
**meristics**	**SL**	**IL**	**PVT**	**LRT**	**VS**	**T4LE**	**T4LU**	**T4TL**	**TFS**	**PS**	**PPS**	**PCT**		
mean	10.38	9.38	33.38	20.13	42.13	6.63	12.00	18.75	28.88	7.38	3.13	2.51		
±sd	0.7440	0.5175	2.2638	0.6409	2.3566	0.7440	0.7559	0.7071	2.4165	1.5980	0.3536	1.0357		
min	9	9	30	19	38	5	11	18	26	5	3	1		
max	11	10	37	21	46	7	13	20	34	9	4	3		
N	8	8	8	8	8	8	8	8	8	8	8	8		
**morphometrics**	**SVL**	**FL**	**TBL**	**AG**	**HL**	**HW**	**HD**	**ED**	**EE**	**SN**	**EN**	**IO**	**EL**	**IN**
mean	1.8585	1.0603	1.1437	1.5222	1.3214	1.1539	0.9337	0.6737	0.7701	0.9109	0.7581	0.6777	0.2946	0.3418
±sd	0.05861	0.01463	0.01153	0.01162	0.00527	0.01282	0.03000	0.01147	0.01681	0.00693	0.02826	0.02548	0.04026	0.03511
min	1.7243	1.0405	1.1302	1.5113	1.3117	1.1355	0.8960	0.6601	0.7469	0.9004	0.6909	0.6311	0.2371	0.2866
max	1.9009	1.0860	1.1603	1.5483	1.3263	1.1750	0.9714	0.6893	0.8038	0.9247	0.7787	0.7102	0.3428	0.4054
N	8	8	8	8	8	8	8	8	8	8	8	8	8	8
Phnom Khpoh														
**meristics**	**SL**	**IL**	**PVT**	**LRT**	**VS**	**T4LE**	**T4LU**	**T4TL**	**TFS**	**PS**	**PPS**	**PCT**		
mean	10.7	9.3	28.7	17.0	40.0	6.7	13.0	19.7	26.0	9.3	3.0	2.3		
sd	0.58	0.58	1.15	1.00	2.65	0.58	0.00	0.58	0.00	0.58	0.00	1.15		
min	10	9	28	16	37	6	13	19	26	9	3	1		
max	11	10	30	18	42	7	13	20	26	10	3	3		
N	3	3	3	3	3	3	3	3	3	3	3	3		
**morphometrics**	**SVL**	**FL**	**TBL**	**AG**	**HL**	**HW**	**HD**	**ED**	**EE**	**SN**	**EN**	**IO**	**EL**	**IN**
mean	1.8421	1.0567	1.1363	1.5025	1.3072	1.1454	0.9266	0.6878	0.7404	0.8945	0.7504	0.6900	0.2695	0.3688
sd	0.09860	0.00346	0.00574	0.00557	0.00859	0.00040	0.02209	0.01171	0.01367	0.00371	0.00774	0.03742	0.06337	0.02250
min	1.72835	1.05317	1.13071	1.49710	1.29886	1.14503	0.90506	0.67638	0.72637	0.89092	0.74284	0.65175	0.20462	0.34579
max	1.90309	1.06008	1.14219	1.50824	1.31603	1.14582	0.94920	0.69977	0.75369	0.89833	0.75830	0.72652	0.33124	0.39074
N	3	3	3	3	3	3	3	3	3	3	3	3	3	3
Phnom Sampeu														
**meristics**	**SL**	**IL**	**PVT**	**LRT**	**VS**	**T4LE**	**T4LU**	**T4TL**	**TFS**	**PS**	**PPS**	**PCT**		
mean	10.7	10.0	29.9	16.0	37.6	6.1	12.7	18.9	26.0	8.0	2.6	1.4		
sd	0.76	0.82	1.07	1.00	2.30	0.38	0.76	0.90	2.08	0.00	0.53	0.53		
min	10	9	28	15	34	6	12	18	24	8	2	1		
max	12	11	31	17	41	7	14	20	29	8	3	2		
N	7	7	7	7	7	7	7	7	7	7	7	7		
**morphometrics**	**SVL**	**FL**	**TBL**	**AG**	**HL**	**HW**	**HD**	**ED**	**EE**	**SN**	**EN**	**IO**	**EL**	**IN**
mean	1.8406	1.0502	1.1300	1.5054	1.2993	1.1328	0.9133	0.6655	0.7311	0.8915	0.7646	0.6706	0.2311	0.3475
sd	0.04343	0.02355	0.01813	0.00650	0.00566	0.00929	0.02377	0.01475	0.02156	0.00573	0.00415	0.02465	0.05812	0.03146
min	1.7513	1.0148	1.1137	1.4952	1.2942	1.1145	0.8739	0.6350	0.6936	0.8835	0.7577	0.6399	0.1326	0.2967
max	1.8887	1.0827	1.1648	1.5150	1.3108	1.1420	0.9501	0.6772	0.7608	0.9022	0.7698	0.7045	0.2956	0.3900
N	7	7	7	7	7	7	7	7	7	7	7	7	7	7

**Table 6.** Data for the referred series of the Phnom Banan population from Phnom Banan, Battambang Province, Cambodia.

Specimen	LSUHC 15174	LSUHC 15173	LSUHC 15170	LSUHC 15169	LSUHC 15171
sex	m	m	f	m	m
**Meristic characters**					
supralabials (SL)	10	9	11	10	11
infralabials (IL)	10	9	10	11	9
paravertebral tubercles (PVT)	37	33	36	38	33
longitudinal rows of tubercles (LRT)	20	19	20	19	18
ventral scales (VS)	39	43	40	45	41
expanded subdigital lamellae on 4^th^ toe (E4TL)	7	8	7	8	7
unmodified subdigital lamellae on 4^th^ toe (U4TL)	16	14	14	15	13
total subdigital lamellae on 4^th^ toe (T4TL)	23	22	21	23	20
enlarged femoral scales (FS) (R,L)	15+14	13+13	10+9	13+14	15+15
total of enlarged femoral scales (TFS)	29	24	19	27	30
femoral pores (FP)	0	0	0	0	0
enlarged precolacal scales (PS)	10	10	12	10	9
precloacal pores (PP)	10	10 1 scale gap	12 dimples	10	9
post-precloacal scale rows (PPS)	3	2	3	3	3
postcloacal tubercles (PCT)	4	3	3	3	3
dark body bands (BB)	4	4	4	4	4
dark caudal bands (DCB)	/	10	/	11	12
light caudal bands (LCB)	/	/	/	10	11
**Categorical characters**					
body tubercles greatly reduced (Tub-red)	no	no	no	no	no
body tubercles moderately keeled (Tub-kld)	yes	yes	yes	yes	yes
caudal tubercles extend beyond base of tail (CT-ext)	yes	yes	yes	yes	yes
subcaudals expanded (SubC-exp)	yes	yes	yes	yes	yes
subccaudals extend up onto lateral side of tail (SubC-lat)	no	no	no	no	no
enlarged femoral and precloacal scales continuous (FS-PS)	yes	yes	no	yes	yes
enlarged proximal femoral < 1/2 size of distal femorals or equal in size (FS-sz)	equal	equal	equal	equal	subequal
pocketing between digits of hind feet (Dig-pok)	no	no	no	no	no
dorsal pattern faded (DP-fad)	yes	yes	slightly	yes	no
distinct dark blotches on top of head present (HD-mrk)	no	no	no	no	no
dark body bands with lightened centers (BB-Cntr)	yes	yes	yes	yes	no
dark body markings in light interspaces (BB-intr)	no	no	no	no	no
dark dorsal bands thin or ~ same width as light interspaces (BB-wd)	wider	wider	wider	wider	wider
light interspaces reduced to a narrow thin white band	no	no	no	no	no
dark dorsal bands bordered by prominently white tubercles (WHT-tub)	yes	yes	yes	yes	yes
dark caudal bands wider than light caudal bands (DCB-wd)	yes	yes	yes	yes	yes
light caudal bands bearing dark marking in adults (WCB-mrk)	yes	yes	yes	yes	yes
**Morphometric characters (mm)**					
SVL	71	74	78.9	73.7	56.7
TL	85	82	88	/	81
TW	6.4	6.1	6.3	6.4	4.5
FL	10.8	12.8	13	13.2	9
TBL	14.9	15.5	15.7	15.7	11.7
AG	32.3	33.4	43.9	33.3	26.2
HL	21.2	21.6	22.6	21.6	16.6
HW	14.1	14.5	14.4	14.6	10.8
HD	8.6	8.7	9	9.2	6.9
ED	4.9	5	5	4.9	4.2
EE	5.6	6	6.1	4.9	4.2
SN	8.3	8.2	8.8	8.3	6.7
EN	6.3	6.3	6.6	6.2	4.9
IO	4.6	5.2	4.5	4.1	3.1
EL	2.2	1.6	2	2.1	1.8
IN	2.3	2.4	2.3	2.2	2

**Table 7.** Data for the referred series Sampeu from the Phnom Sampeu and Phnom Khpoh populations, Battambang Province, Cambodia.

Locality	Phnom Sampeu	Phnom Sampeu	Phnom Sampeu	Phnom Sampeu	Phnom Sampeu	Phnom Sampeu	Phnom Sampeu	Phnom Khpoh	Phnom Khpoh	Phnom Khpoh
specimen	LSUHC 15113	LSUHC 15115	LSUHC 15116	LSUHC 15118	LSUHC 15114	LSUHC 15117	LSUHC 15120	LSUHC 15230	LSUHC 15229	LSUHC 15231
sex	m	m	f	m	m	m	f	f	f	m
**Meristic characters**										
supralabials (SL)	12	11	10	11	11	10	10	11	10	11
infralabials (IL)	10	9	9	10	10	11	11	10	9	9
paravertebral tubercles (PVT)	30	30	31	31	29	28	30	28	28	30
longitudinal rows of tubercles (LRT)	15	15	17	16	17	15	17	16	18	17
longitudinal rows of ventral scales (VS)	36	39	37	34	39	41	37	37	42	41
expanded subdigital lamellae on 4^th^ toe (E4LT)	6	7	6	6	6	6	6	6	7	7
unmodified subdigital lamellae on 4^th^ toe (U4LT)	12	13	12	14	13	12	13	13	13	13
total subdigital lamellae on 4^th^ toe (T4TL)	18	20	18	20	19	18	19	19	20	20
enlarged femoral scales (R,L)	13+11	13+13	16+13	15+13	11+13	12+12	15+12	15+11	16+10	14+12
total of enlarged femoral scales (TFS)	24	26	29	28	24	24	27	26	26	26
femroal pores (FP)	0	0	8 dimples	0	0	0	8 weak dimples	0	0	0
enlarged precolacal scales (PS)	8	8	8	8	8	8	8	10	9	9
precloacal pores (PP)	8	8	8 small	8	8	8	8 small	10 dimples	9 dimples	9 dimples
post-precloacal scale rows (PPS)	2	3	2	3	2	3	3	3	3	3
postcloacal tubercles (PCT)	2	2	1	1	1	2	1	3	3	1
body dark bands (BB)	4	4	4	4	4	4	4	4	4 on sacrum	4
dark caudal bands (DCB)	10	11	10	/	/	/	/	10	/	/
light caudal bands (LCB)	9	10	10	/	/	/	/	10	/	/
**Categorical characters**										
body tubercles greatly reduced (Tub-red)	no	no	no	no	no	no	no	no	no	no
body tubercles moderately keeled (Tub-kld)	yes	yes	yes	yes	yes	yes	yes	yes	yes	yes
caudal tubercles extend beyond base of tail (CT-ext)	yes	yes	yes	yes	yes	yes	yes	yes	yes	yes
subcaudals expanded (SubC-exp)	yes	yes	yes	yes	yes	yes	yes	yes	yes	yes
subccaudals extend up onto lateral side of tail (SubC-lat)	no	no	no	no	no	no	no	no	no	no
enlarged femoral and precloacal scales continuous (FS-PS)	yes	yes	yes	yes	yes	yes	yes	no	no	no
proximal femoral < 1/2 size of distal femorals (FS-sz)	equal	equal	yes	yes	equal	yes	yes	yes	yes	yes
pocketing between digits of hind feet (Dig-pok)	no	no	no	no	no	no	no	no	no	no
dorsal pattern faded (DP-fad)	yes	yes	yes	yes	yes	yes	yes	no	no	no
distinct dark pigmented blotches on top of head present (HD-mrk)	no	no	no	no	no	no	no	no	faint	no
dark body bands with lightened centers (BB-Cntr)	yes	yes	yes	yes	yes	yes	yes	yes	yes	yes
dark body markings in light interspaces (BB-intr)	no	no	no	no	no	no	no	slightly	slightly	slightly
dark dorsal bands thin or ~ same width as light interspaces (BB-wd)	equal	equal	equal	equal	slightly wider	slightly wider	slightly wider	wider	equal	wider
light interspaces reduced to a narrow thin white band (INT-red)	no	no	no	no	no	no	no	no	no	no
dark dorsal bands bordered by prominently colored white tubercles (WHT-tub)	yes	yes	yes	yes	yes	yes	yes	yes	yes	yes
dark caudal bands wider than light caudal bands (DCB-wd)	yes	yes	yes	yes	yes	yes	yes	yes	yes	yes
light caudal bands bearing dark marking in adults (WCB-mrk)	yes	yes	yes	yes	yes	yes	yes	faint	faint	faint
**Morphometric characters (mm)**										
SVL	72.4	70.7	80.0	71.2	71.8	67.1	56.4	80.0	78.5	53.5
TaiL	100.0	88.0	104.0	75.0	88.0	75.0	73.0	81.0	77.0	58.0
TaiW	6.5	5.3	6.2	5.8	6.2	5.5	4.8	6.3	6.3	4.6
FL	12.2	11.9	11.6	12.4	11.4	10.4	8.8	13.3	12.8	8.2
TBL	15.2	13.2	14.6	14.2	13.6	12.7	11.0	15.4	15.5	10.2
AG	33.6	32.6	37	32.7	32.9	30	25.5	36.5	36.6	22.7
HL	21.2	20.1	21.7	20.1	20.6	19.2	16.5	22.3	22.8	15.7
HW	14.5	13.7	15.4	14.1	14.0	12.5	10.9	16.0	15.7	10.3
HD	8.4	7.6	9.0	8.7	8.6	8.6	6.5	8.9	9.7	6.7
ED	4.9	4.7	5.2	4.4	4.7	4.6	3.9	5.3	5.5	3.8
EE	5.1	5.4	6.1	5.6	5.4	5.6	4.5	6.4	5.9	4.2
SN	8.0	7.9	8.5	7.8	8.2	7.6	6.5	8.7	8.7	6.1
EN	6.1	5.9	6.3	6.0	6.0	5.6	4.8	6.3	6.4	4.2
IO	4.9	4.7	4.6	4.4	5.0	5.0	4.2	6.2	5.1	3.5
EL	1.4	2.0	2	1.7	1.6	1.9	1.4	2.3	1.7	1.6
IN	2.4	2.0	2.3	2.2	2.4	2.4	1.9	2.7	2.4	1.9

In meristics, *Cyrtodactyluskampingpoiensis* sp. nov. differs significantly from the P. Sampeu population in having more rows of longitudinal tubercles (LRT: 19–21 vs 15–17). It differs significantly from the P. Khpoh population and the P. Sampeu population in having a greater number of paravertebral tubercles (PVT 30–37 vs 28–30 and 28–31, respectively) (Tables 3–5). It differs significantly morphometrically from the P. Sampeu population in having a significantly shorter ear-eye distance (EE) and a wider and longer head (HL and HW, respectively) and from the P. Sampeu and P. Khpoh populations in having a longer snout (SN) and longer hind limbs (TBL) (Table 3). Categorically it differs from the P. Sampeu population in having discontinuous versus continuous enlarged femorals and precloacals (FS-PS) and there are no dark markings in the light interspaces between the dark body bands (BB-intr) whereas faint markings occur in the P. Khpoh population (Figs 7, 9, 10; Suppl. material 3).

##### Natural history.

**Figure 8.**
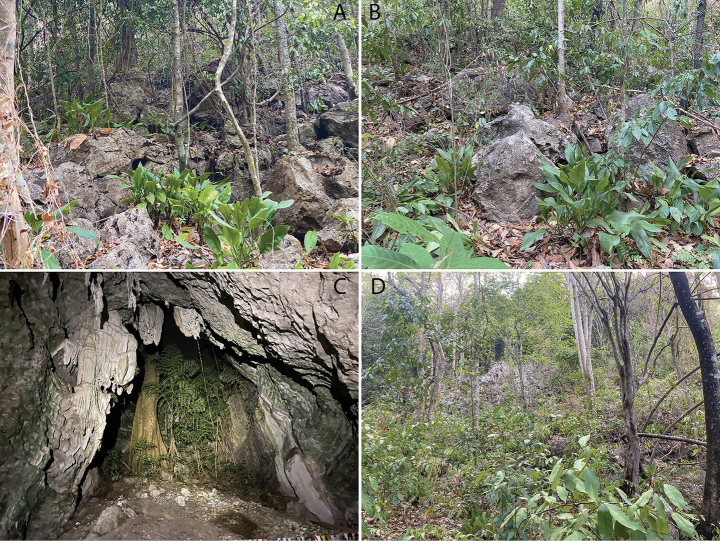
Habitat at Phnom Kamping Poi **A, B** optimal microhabitat of boulders of varying size within the karst vegetation **C** cave microhabitat inside Damrei Cave **D** typical deciduous karst vegetation.

All specimens of the type series were collected on P. Kamping Poi from 1030–2000 hrs. Phnom Kamping Poi is a long rectangularly shaped karstic hill ca 6.5 km in length and 1.6 wide and its widest point near its southeastern margin (Fig. 1). Phnom Kamping Poi reaches ca 242 m in elevation and is covered with drought-deciduous karst vegetation (Fig. 8). The hillsides are covered with karstic boulders of varying size which constitutes the prime microhabitat for *C.kampingpoiensis* sp. nov. although some specimens were found in a wide, open cave near the entrance. All age classes from hatchlings (LSUHC 15198; 39 mm SVL) to adults (LSUHC 15206, 15209, 15212; 82 mm SVL) were found and LSUHC 15207 (SVL 77.9 mm) was gravid with two eggs. Although geckos were found on all substrates, they were most commonly found on karst in all planes of orientation with fewer specimens being found on the ground and the karst vegetation – often at the base of trees. One specimen (LSUHC 15204 [in the FFI collection]) was found deep within a cave on top of a large (2.5 m diameter) log. Other species found on P. Kamping Poi were the lizards *Eutropislongicaudata* (Hallowell), *Dixoniussiamensis* (Boulenger), *Gekkogecko* (Linnaeus), Gehyracf.lacerata (Taylor), *Gehyramutilata* (Wiegmann) *Hemidactylusfrenatus* Duméril & Bibron, and *Subdolosepsbowringii* (Günther), and the snakes *Lycodoncapucinus* Boie and Indotyphlopscf.braminus (Daudin).

**Figure 9.**
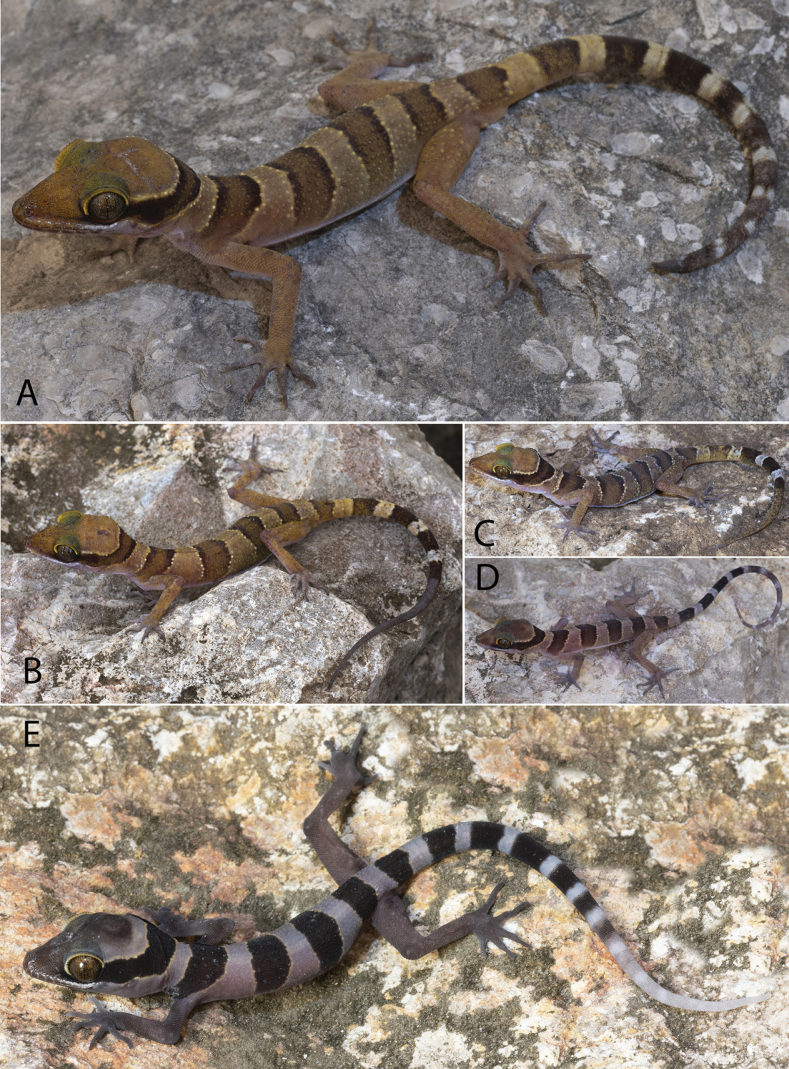
The Phnom Banan population **A** adult male LSUHC 15173 **B** adult female LSUHC 15168 in FFI collection **C** adult female LSUHC 15170 **D** juvenile LSUHC 15176 **E** hatchling LSUHC 15175.

**Figure 10.**
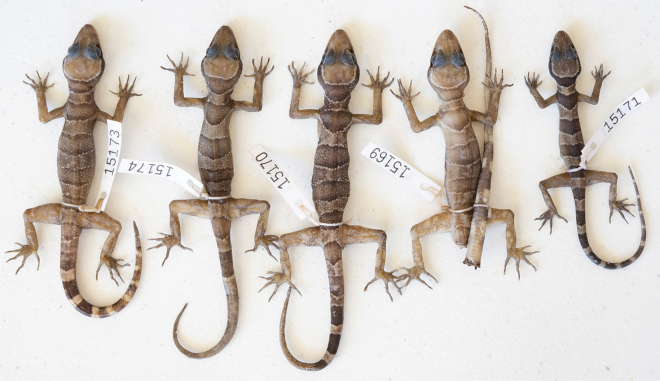
Specimens from the Phnom Banan population.

**Figure 11.**
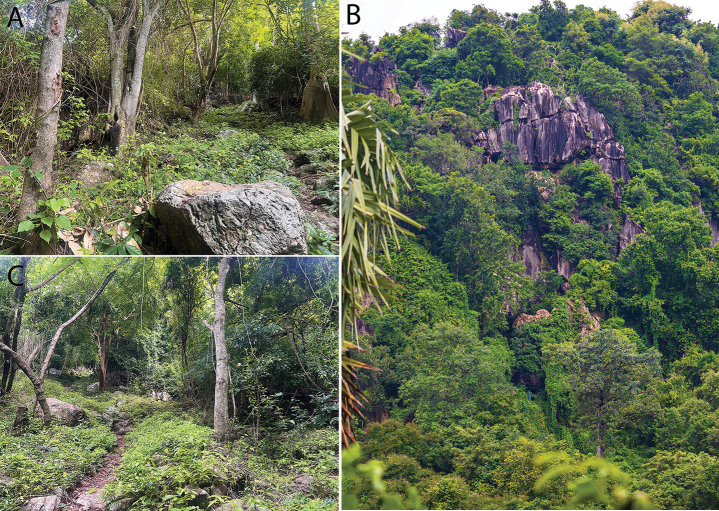
Habitat at Phnom Banan **A** hillside microhabtiat **B** Cliff face microhabitat **C** deciduous karst vegetation following summer rains. Photographs by Sothearen Thi.

All specimens from P. Banan (Figs 9, 10) were collected near Rum Say Sok from 1930–2000 hrs. Phnom Banan is an irregularly shaped elongate karstic hill ca 6.6 km in length and 1.6 km wide at its widest point near its the eastern margin (Fig. 1). It reaches ca 203 m in elevation and is covered with drought-deciduous karst vegetation (Fig. 11). All age classes from hatchlings (LSUHC 15177 [in FFI collection]; 35.0 mm SVL) to adults (LSUHC 15168 [in FFI collection]; SVL 82.0 m) were found, indicating that mid-March is within the reproductive season for this population. Although geckos were occasionally found on the ground (most hatchings and juveniles), they were most commonly found on the karst surfaces in all planes of orientation with fewer specimens being found on karst vegetation (only at the base of trees less than 0.5 m above the ground or on fallen logs). Other species found on P. Banan were the frogs Polypedatescf.leucomystax (Gravenhorst); *Glyphoglossusguttulatus* (Blyth); *Kaloulapulchra* Gray; *Microhylamukhlesuri* Hasan, Islam, Kuramoto, Kurabayashi & Sumida; the lizards *Gehyramutilata* (Wiegmann); *Subdolosepsbowringii* (Günther); and *Eutropismacularia* (Blyth); and the snakes Oligodoncf.kampucheaensis Neang, Grismer & Daltry; and a *Chrysopeleaornata* (Shaw) found dead on a nearby road.

**Figure 12.**
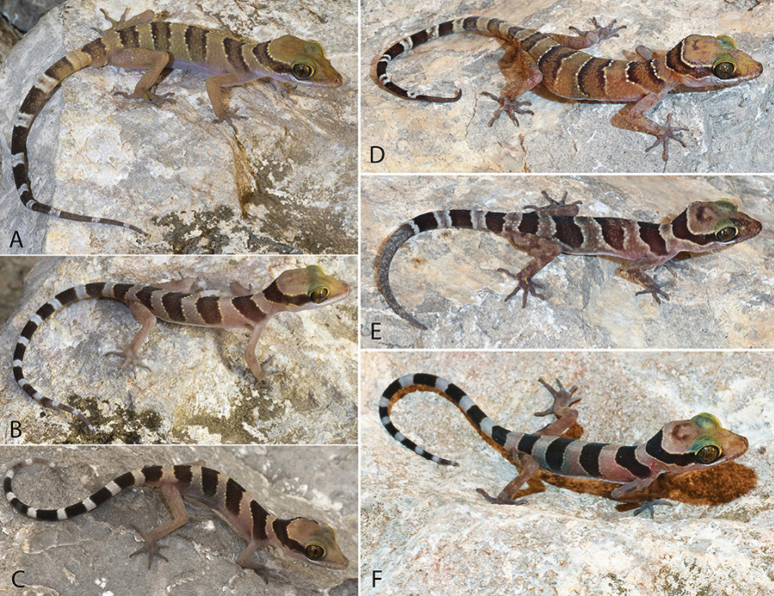
**A, C** The Phnom Sampeu population **A** adult male LSUHC 15113 **B** juvenile LSUHC 15119 in FFI collection **C** hatchling LSUHC 15123 **D–F** the Phnom Khpoh population **D** gravid adult female LSUHC 15229 **E** juvenile female LSUHC 15231 **F** hatchling LSUHC 15233 in FFI collection.

**Figure 13.**
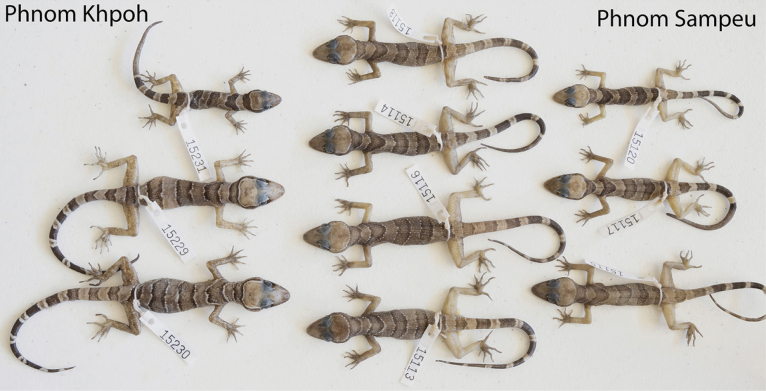
Comparison of specimens from the Phnom Khpoh and Phnom Sampeu populations.

**Figure 14.**
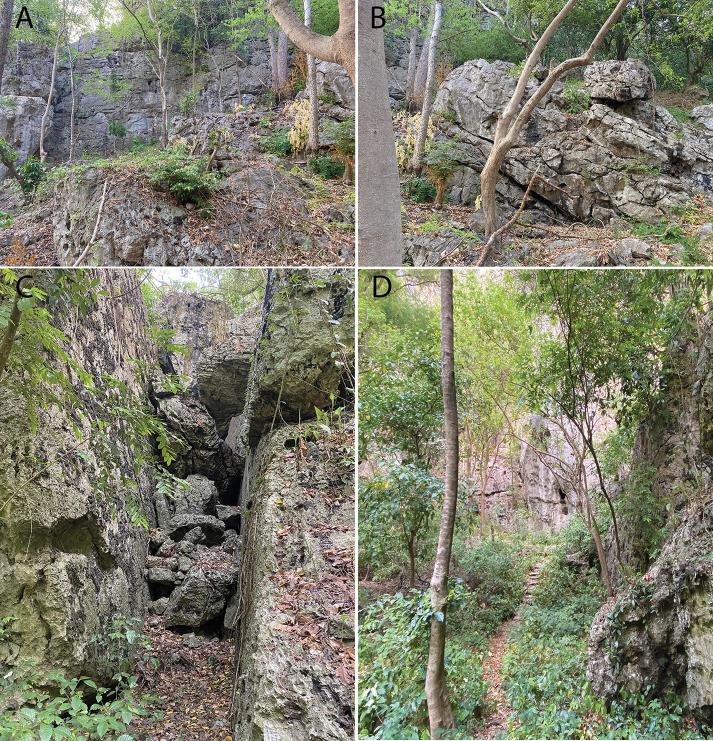
Habitat at Phnom Sampeu **A** vertical face microhabitat of a low karstic ridge **B** boulder microhabitat within open karst vegetation **C** vertical microhabitat **D** typical deciduous karst vegetation where LSUHC 15118 was found on the base of the tree in the foreground.

All specimens were from P. Sampeu (Figs 12, 13) from were collected from 1930–2050 hrs. Phnom Sampeu is a small oval karstic hill ca 1.8 km in length and 0.6 km across the center (Fig. 1). It reaches 102 m in elevation and is covered with drought-deciduous karst vegetation. The hillsides are covered with karstic boulders of varying size as well as shear vertical faces where specimens were most commonly found (Fig. 14). Geckos, however, were also found on the ground and on karst vegetation, usually at the base of trees. All age classes from hatchlings (LSUHC 15126; 35 mm SVL) to adults (LSUHC 151116; 80 mm SVL) were observed, indicating mid-March is within the reproductive season of the P. Sampeu population. Specimen LSUHC 15113 had eaten a large Huntsman Spider. Other species found on P. Sampeu were the frogs Duttaphrynuscf.melanostictus (Schneider), *Kaloulapulchra* Gray, and Polypedatescf.leucomystax (Gravenhorst), the lizards *Dixoniussiamensis* (Boulenger), and the snakes *Lycodoncapucinus* Boie and Trimeresuruscf.albolabris Gray.

**Figure 15.**
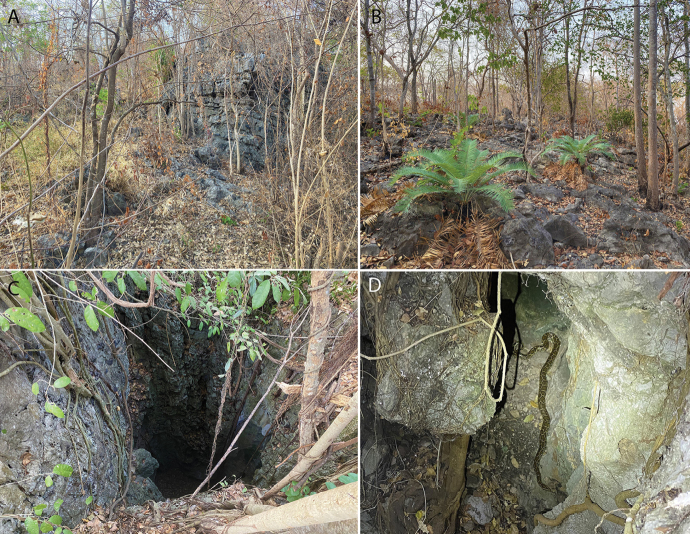
Habitat at Phnom Khpoh **A** typical boulder microhabitat within karst vegetation **B** open rocky microhabitat where specimens are rarely found **C** vertical opening into a cave microhabitat **D** cave microhabitat shared with *Pythonbivittatus*.

Phnom Khpoh is overall a somewhat circularly shaped karstic hill with deeply incised margins 8.6 km to the west of P. Sampeu. It is 5.0 km by 4.5 km in size with three major peaks – the western and eastern peaks reaching ca 250 m in elevation and a southern peak reaching ca 242 m in elevation (Fig. 1). Like the other hills, P. Khpoh is covered with drought-deciduous karst vegetation, varying karst boulder microhabitats, and caves of varying length and depth (Fig. 15). The three specimens collected were found on karst and vegetation from 1900–2030 hrs at the opening of a small cave. A fourth specimen was found low on a cave wall ~ 4 m from the opening (Figs 13, 15). All age classes from hatchlings (LSUHC 15232 [in the FFI collection; 36 mm SVL]) to adults (LSUHC 15230; 80.0 mm SVL) were observed. LSUHC 15229 (SVL 78.5 mm) is gravid. Other species found on P. Khpoh were the frogs Duttaphrynuscf.melanostictus (Schneider), *Kaloulapulchra* Gray, and Polypedatescf.leucomystax (Gravenhorst), the lizards *Dixoniussiamensis* (Boulenger) and *Hemiphyllodactyluskhpoh* Grismer, Sinovas, Quah, Thi, Chourn, Chhin, Hun, Cobos, Geissler, Ching & Murdoch, and the snakes *Lycodoncapucinus* Boie and Trimeresuruscf.albolabris Gray.

## ﻿Discussion

### ﻿*Cyrtodactylus* diversity in Cambodia

**Figure 16.**
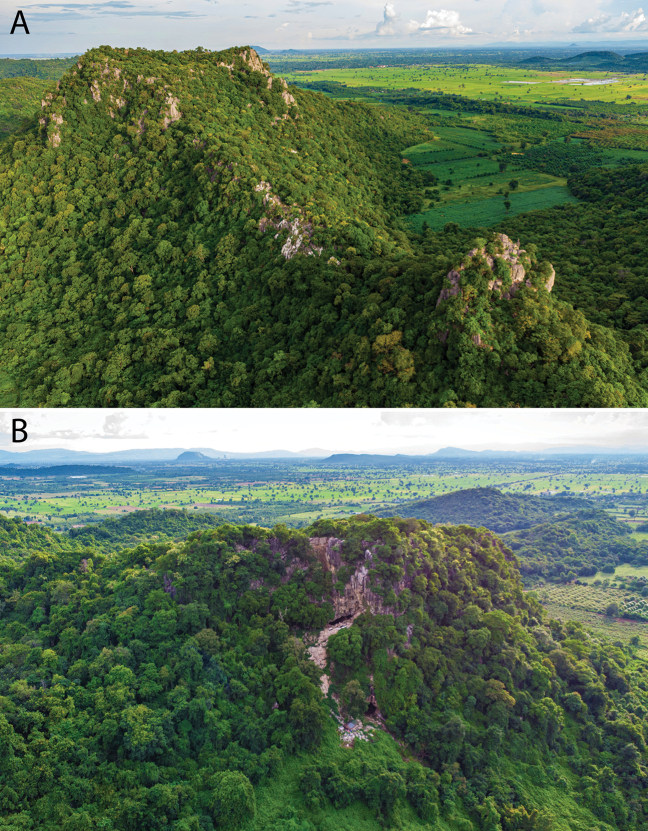
**A** Karstic ridge of Phnom Banan looking east **B** west end of Phnom Banan looking north. A cave opening can be observed near the summit. Several unsurveyed karst hills cab be seen in the background on both photographs. Drone photographs taken by Phyroum Chourn.

The description of *Cyrtodactyluskampingpoiensis* sp. nov. brings the total number of named *Cyrtodactylus* in Cambodia to 10. Much of this diversity was due to the partitioning of *C.intermedius* into seven species (Murdoch et al. 2019) followed by descriptions of additional species (see summary in Chhin et al. 2024). However, given that the neighboring countries of Thailand, Laos, and Vietnam have 55, 25, and 55 named species, respectively, is a clear indication that *Cyrtodactylus* diversity in Cambodia is underestimated, even given its smaller size than the former countries. This is due mostly to the fact that large areas of Cambodia remain completely unexplored – especially areas with habitats such as karst which are known to harbor an inordinately large number of specialized karst-dwelling *Cyrtodactylus* (Grismer et al. 2021b). Western Cambodia is just such an area. Our survey covered only seven karstic outcroppings in a small area (Fig. 1), but we observed five separate populations, at least four of which for now represent a new species. During a reconnaissance of the region between the cities of Battambang and Palin near the Thai-Cambodia border, a straight-line distance of only ca 62 km, we observed ~ 30 unexplored karstic areas ranging from small towers to mountain ranges (Fig. 16). There are also unexplored karstic areas in northern and southern Cambodia. The discovery of this new species and its considerable degree of morphological interpopulational variation underscores the necessity for further exploration in order to begin understanding the overall herpetological diversity of Cambodia in general, and western Cambodia in particular, where dozens of isolated karstic habitats still remain to be surveyed. Given the extremely localized distribution of these four new populations, each meets the IUCN criteria of distinct genetic lineages that are Critically Endangered.

### ﻿Morphometrics versus meristics

These data sets were analyzed using separate PCAs because each provides a different type of information concerning the morphological evolution and adaptation of these populations as well as different diagnostic systems. Even though all populations plot separately in both PCAs (Figs 3, 4) this does not mean they are all significantly different from one another in both analyses. The PERMANOVA and DAPC of the morphometric data clearly demonstrate that body shape among these populations is far more conserved than the plots illustrate, being that only two population pairs differ significantly from one another (Fig. 3B, C). Whereas the PERMANOVA and DAPC for the meristic data indicate that nearly all the populations differ significantly from one another and all but one of these species pairs differed at the more rigorous *p*-adjusted level (Fig. 4B, C). This would suggest that body shape for living in a highly specialized karstic microhabitat is under stronger selection pressure and as such, is able to vary less whereas the number of scales on various parts of the body can vary randomly and may be under no selection at all. A number of studies have shown that karst-dwelling *Cyrtodactylus* are morphometrically different than species living on other substrates and may even differ among species living on different kinds of karst (e.g., Chomdej et al. 2020; Grismer et al. 2020a, b, 2021a, 2023b; 2024a; Kaatz et al. 2021; Termprayoon et al. 2023; Wood et al. 2020). Understanding these differences can aid in putting into place more effective and efficient conservation measures.

### ﻿One, three, or four species?

The advent of integrative taxonomy with its incorporation of molecular phylogenies, has been instrumental in recovering unrealized species diversity within species complexes. Molecular analyses offer a more fine-grained, less subjective approach to species delimitation that is divorced from a strictly morphological approach which diagnoses and delimits species simultaneously. An inherent error with conflating these independent operations (see Frost and Hillis 1990; Frost and Kluge 1994; Hillis 2019) is the assumption that “diagnosable” populations lack gene flow – usually the primary criterion for delimiting species (de Queiroz 2007). Commonly exacerbating this issue, are morphological descriptions lacking statistical defensibility (see Chan and Grismer 2022), leaving any assumption of interspecific gene flow weak, thus rendering the validity of the “new” species dubious, typical of late 19^th^ and early 20^th^ century taxonomy (see for example Pauwels et al. 2024; Sumontha et al. 2024). We are aware that species descriptions based on older museum material do not avail themselves to molecular data, which is all the more reason, when possible, to provide rigorous statistical diagnoses.

We elect to recognize *Cyrtodactyluskampingpoiensis* sp. nov. as a new geographically variable species. Even though the mitochondrial phylogeny may indicate there is no interpopulational gene flow – given that all the populations are monophyletic – and it aligns with their statistically significant and categorical diagnosability, the presence or absence of gene flow requires definitive confirmation with nuclear markers – preferably a genomic data set – especially when genetic divergence is low. Notably, these newly discovered populations are allopatric on separate karst formations separated by ca 5–20 km of uninhabitable terrane (mostly degraded savannah or paddy fields) indicating that migration among these formations is extremely unlikely, particularly true of karst-adapted geckos with low vagility. Furthermore, unlike oceanic islands where passive overwater dispersal can result in admixture similar to upland sky-island habitats and land bridge islands that form and reform with glacial cycling, separate karst formations do not reform. In general, the separate towers, cones, and hills become isolated through erosion, weathering, and changing water courses (Wang et al. 2019). Thus, species adapted to live only in karstic microhabitats are essentially restricted to these environs. This is not to say that a habitat generalist may not disperse into a karstic area and hybridize with a karst-dwelling species, but we see no evidence of this in *Cyrtodactylus* even where karst-dwelling species and species with different habitat preferences are syntopic on the periphery of the karst formation (e.g., *C.astrum* [karst] and *C.macrotuberculatus* [granite forest] in Peninsular Malaysia; Grismer 2011; Grismer et al. 2012). Nonetheless, until the absence of gene flow is unequivocally confirmed, we reluctantly elect to consider all four populations to be a single geographically variable species. We realize this decision may not be in the true spirit of “integrative taxonomy” (Padial et al. 2010) that aims to integrate all forms of relevant data – geogrpahy, geology, ecology, and natural history, and that perhaps genetic distance alone should *not* be the overriding factor. We are currently working on a genomic analysis of these populations.

### ﻿Conservation

The high levels of biodiversity and site-specific endemism in many karstic regions rival that of most other habitats, yet these regions are rapidly becoming some of the most imperiled ecosystems in Southeast Asia (Clements et al. 2006; [Bibr B30][Bibr B56]; Quah et al. 2021). Southeast Asia harbors more karst habitat than anywhere else in the world (Day and Urich 2000) but unregulated and unsustainable quarrying practices, the primary threat to karst-dwelling species, continues to degrade the integrity of these landscapes in many developing countries where enormous monetary gains override issues of conservation. This only serves to amplify the biodiversity crisis in Southeast Asia, where the overall rate of habitat loss is the highest among the world’s tropical regions (Sodhi and Brook 2006). A number of studies have shown that there are far more karst-associated vertebrates in Southeast Asia than previously reported (e.g., Luo et al. 2016; Connette et al. 2017) and the discovery rate of new species of karst-adapted amphibians and reptiles shows no signs of leveling off. Continued field surveys in western Cambodia will no doubt add to this increasing rate. The report here, and that of Grismer et al. (2025) of the new species of site-restricted, karst-dwelling *Hemiphyllodactyluskhpoh* from Battambang Province alone, underlines the conservation and management needs of these areas in Cambodia. Given the extremely localized distribution of these newly discovered species and populations, each meets the IUCN criteria of being Critically Endangered. Some of the karst formations harboring site-specific endemics are also religious retreats and protected places of Buddhist worship, and thus under some level of *de facto* protection from quarrying operations. However, dozens are not and, as such, they remain under the threat of unsustainable quarrying. Intensive biodiversity systematic surveys of all these formations is paramount to underpin measures of protection for this portion of Cambodia’s natural heritage.

## Supplementary Material

XML Treatment for Cyrtodactylus kampingpoiensis

## ﻿Additional information

### Author ORCIDs

Evan S. H. Quah https://orcid.org/0000-0002-5357-1953

L. Lee Grismer https://orcid.org/0000-0001-8422-3698

Pablo Sinovas https://orcid.org/0000-0001-5396-8910

Phyroum Chourn https://orcid.org/0009-0005-8664-5321

Sophea Chhin https://orcid.org/0000-0002-5612-7532

Seiha Hun https://orcid.org/0009-0004-0147-1565

Anthony Cobos https://orcid.org/0009-0001-9215-4052

Peter Geissler https://orcid.org/0000-0001-7408-1358

Christian Ching https://orcid.org/0000-0001-6972-6836

Matthew L. Murdoch https://orcid.org/0000-0001-5914-6408

Sothearen Thi https://orcid.org/0009-0009-4827-1719

Jesse L. Grismer https://orcid.org/0000-0002-2542-5430

### Data availability

All of the data that support the findings of this study are available in the main text or Supplementary Information.

## Supplementary materials

Supplementary material 1 Data used in the MFA and PCA analyses Data type xlsx https://binary.pensoft.net/file/1344957This dataset is made available under the Open Database License (http://opendatacommons.org/licenses/odbl/1.0/). The Open Database License (ODbL) is a license agreement intended to allow users to freely share, modify, and use this Dataset while maintaining this same freedom for others, provided that the original source and author(s) are credited.Evan S. H. Quah, L. Lee Grismer, Pablo Sinovas, Phyroum Chourn, Sophea Chhin, Seiha Hun, Anthony Cobos, Peter Geissler, Christian Ching, Matthew L. Murdoch, Sothearen Thi, Jeren J. Gregory, Eddie Nguyen, Alexis P. Hernandez, Amanda Kaatz, Jesse L. Grismer

Supplementary material 2 Uncorrected pariwise sequence divergences based on 1,035 base pairs of ND2 bewteen the Batambang clade and the *intermedius* group Data type xlsx https://binary.pensoft.net/file/1344958This dataset is made available under the Open Database License (http://opendatacommons.org/licenses/odbl/1.0/). The Open Database License (ODbL) is a license agreement intended to allow users to freely share, modify, and use this Dataset while maintaining this same freedom for others, provided that the original source and author(s) are credited.Evan S. H. Quah, L. Lee Grismer, Pablo Sinovas, Phyroum Chourn, Sophea Chhin, Seiha Hun, Anthony Cobos, Peter Geissler, Christian Ching, Matthew L. Murdoch, Sothearen Thi, Jeren J. Gregory, Eddie Nguyen, Alexis P. Hernandez, Amanda Kaatz, Jesse L. Grismer

Supplementary material 3 Inter- and intraspecific variation in categorical characters among populations of the Battambang clade Data type xlsx https://binary.pensoft.net/file/1344959This dataset is made available under the Open Database License (http://opendatacommons.org/licenses/odbl/1.0/). The Open Database License (ODbL) is a license agreement intended to allow users to freely share, modify, and use this Dataset while maintaining this same freedom for others, provided that the original source and author(s) are credited.Evan S. H. Quah, L. Lee Grismer, Pablo Sinovas, Phyroum Chourn, Sophea Chhin, Seiha Hun, Anthony Cobos, Peter Geissler, Christian Ching, Matthew L. Murdoch, Sothearen Thi, Jeren J. Gregory, Eddie Nguyen, Alexis P. Hernandez, Amanda Kaatz, Jesse L. Grismer

Supplementary material 4 Selected potential diagnostic characters of the morphological, meristic, and catagorical data among the species *Cyrtodactylusintermedius* group Data type xlsx https://binary.pensoft.net/file/1344960This dataset is made available under the Open Database License (http://opendatacommons.org/licenses/odbl/1.0/). The Open Database License (ODbL) is a license agreement intended to allow users to freely share, modify, and use this Dataset while maintaining this same freedom for others, provided that the original source and author(s) are credited.Evan S. H. Quah, L. Lee Grismer, Pablo Sinovas, Phyroum Chourn, Sophea Chhin, Seiha Hun, Anthony Cobos, Peter Geissler, Christian Ching, Matthew L. Murdoch, Sothearen Thi, Jeren J. Gregory, Eddie Nguyen, Alexis P. Hernandez, Amanda Kaatz, Jesse L. Grismer

## References

[B1] Abzhanov A (2010) Darwin’s Galapagos finches in modern biology. Philosophical Transactions of the Royal Society B 365: 1001–1007. 10.1098/rstb.2009.0321 PMC2830240 20194163

[B2] Ampai N Rujirawan A Yodthong S Termprayoon K Stuart BL Aowphol A (2024) A new species of karst-dwelling bent-toed gecko of the *Cyrtodactylusintermedius* group (Squamata, Gekkonidae) from eastern Thailand and the phylogenetic placement of *C.intermedius*. ZooKeys 1211: 101–130. 10.3897/zookeys.1211.122563 PMC11387836 39262607

[B3] Barraclough TG Birky Jr CW Burt A (2003) Diversification in sexual and asexual organisms. Evolution 57: 2166–2172. 10.1111/j.0014-3820.2003.tb00394.x 14575336

[B4] Bell RC Parra JL Tonione M Hoskin CJ MacKenzie JB Williams SE Moritz C (2010) Patterns of persistence and isolation indicate resilience to climate change in montane rainforest lizards. Molecular Ecology 19: no–455. 10.1111/j.1365-294X.2010.04676.x 20497322

[B5] Bell RC MacKenzie JB Hickerson MJ Chavarría KL Cunningham M Williams S Mortitz C (2011) Comparative multi-locus phylogeography confirms multiple vicariance events in co-distributed rainforest frogs. Proceedings of the Royal Society B Biological Series 279: 991–999. 10.1098/rspb.2011.1229 PMC3259917 21900325

[B6] Bleeker P (1860) Reptili.n van Agam aangeboden door E. W. A. Ludeking. Natuurkundig Tijdschrift voor Natuurkundige Vereeniging in Nederladsch Indi Batavia 20: 325–329. [In Dutch]

[B7] Camargo A (2022) PCAtest: Testing the statistical significance of principal component analysis in R. PeerJ 10: e12967. 10.7717/peerj.12967 PMC8858582 35194531

[B8] Chan KO Grismer LL (2022) GroupStruct: An R package for allometric size correction. Zootaxa 5124: 471–482. 10.11646/zootaxa.5124.4.4 35391110

[B9] Chhin S Neang T Chan S Kong K Ou R Samorn V Sor R Lou V Sin S Chhim M Stuart BL Grismer LL (2024) A new species in the *Cyrtodactylusintermedius* (Squamata: Gekkonidae) group from an isolated limestone karst formation in southwestern Cambodia. Zootaxa 5474: 1–20. 10.11646/zootaxa.5474.1.1 39646510

[B10] Chomdej S Suwannapoom C Pawangkhanant P Pradit W Nazarov RA Grismer LL Poyarkov NA (2020) A new species *Cyrtodactylus* Gray (Squamata: Gekkonidae) from western Thailand and the phylogenetic placement of *C.inthanon* and *C.doisuthep*. Zootaxa 4838: 179–209. 10.11646/zootaxa.4838.2.2 33056821

[B11] Clements R Sodhi NS Schilthuizen M Ng PKL (2006) Limestone karsts of Southeast Asia: imperiled arks of biodiversity. BioScience 56: 733–742. 10.1641/0006-3568(2006)56[733:LKOSAI]2.0.CO;2

[B12] Connette GM Oswald P Thura MK Connette KJL Grindley ME Songer M Zug GR Mulchay DG (2017) Rapid forest clearing in a Myanmar proposed national park threatens two newly discovered species of geckos (Gekkonidae: *Cyrtodactylus*). PLOS ONE 12: e0174432. 10.1371/journal.pone.0174432 PMC5389631 28403189

[B13] Darwin C (1859) On the origin of species by means of natural selection, or, The preservation of favoured races in the struggle for life. J. Murray, London. 10.5962/bhl.title.68064 PMC5184128 30164232

[B14] Day M Urich P (2000) An assessment of protected karst landscapes in Southeast Asia. Cave and Karst Science 27: 61–70.

[B15] de Queiroz K (2007) Species concepts and species delimitation. Systematic Biology 56: 879–886. 10.1080/10635150701701083 18027281

[B16] de Queiroz K (2011) Branches in the lines of descent: Charles Darwin and the evolution of the species concept. Biological Journal of the Linnean Society 103: 19–35. 10.1111/j.1095-8312.2011.01634.x

[B17] de Queiroz K (2020) An updated concept of subspecies resolves a dispute about the taxonomy of incompletely separated lineages. Herpetological Review 51: 459–461.

[B18] Diamond JM (1977) Continental and insular speciation in Pacific land birds. Systematic Biology 26: 263–268. 10.1093/sysbio/26.3.263

[B19] Drummond AJ Ashton B Buxton S Cheung M Cooper A Duran C Field M Heled J Kearse M Markowitz S Moir R Stones-Havas S Sturrock S Thierer T Wilson A (2011) Geneious. Version 5.6. http://www.geneious.com/ [accessed 9 January 2018]

[B20] Frost DR Hillis DM (1990) Species in concept and practice: herpetological application. Herpetologica 46: 87–104. 10.1655/08-031R1.1

[B21] Frost DR Kluge AG (1994) A consideration of the epistemology in systematic biology, with special reference to species. Cladistics 10: 259–294. 10.1111/j.1096-0031.1994.tb00178.x

[B22] Grant PR Grant BR (2002) Adaptive Radiation of Darwin's Finches Recent data help explain how this famous group of Galápagos birds evolved, although gaps in our understanding remain. Science 90: 130–139. 10.1511/2002.2.130

[B23] Gray JE (1827) A synopsis of the genera of the saurian reptiles, in which some new genera are indicated, and the others reviewed by actual examination. Philosophical Magazine, London, (Ser.2) 2(7): 54–58. 10.1080/14786442708675620

[B24] Grismer LL (2011) Lizards of Peninsular Malaysia, Singapore and their adjacent archipelagos. Edition Chimaira, Frankfurt am Main, 728 pp.

[B25] Grismer LL Grismer JG (2017) A re-evaluation of the phylogenetic relationships of the *Cyrtodactyluscondorensis* group (Squamata; Gekkonidae) and a suggested protocol for the characterization of rock-dwelling ecomorphology in *Cyrtodactylus*. Zootaxa 4300: 486–504. 10.11646/zootaxa.4300.4.2

[B26] Grismer LL Wood JR PL Myint Kyaw Thura Thaw Zin Quah ESH Murdoch ML Grismer MS Aung Lin Htet Kyaw Ngwe Lwin (2018b) Twelve new species of *Cyrtodactylus* Gray (Squamata: Gekkonidae) from isolated limestone habitats in east- central and southern Myanmar demonstrate high localized diversity and unprecedented microendemism. Zoological Journal of the Linnean Society 182: 862–959. 10.1093/zoolinnean/zlx057

[B27] Grismer LL Wood JR PL Myint Kyaw Thura Quah ESH Murdoch ML Grismer MS Herr MW Aung Lin Htet Kyaw (2018c) Three more new species of *Cyrtodactylus* (Squamata: Gekkonidae) from the Salween Basin of eastern Myanmar underscore the urgent need for the conservation of karst habitats. Journal of Natural History 52: 1243–1294. 10.1080/00222933.2018.1449911

[B28] Grismer LL Wood Jr PL Quah ESH Shahrul A Muin MA Sumontha M Norhayati A Bauer AM Wangkulangkul S Grismer JL Pauwels OSG (2012) A phylogeny and taxonomy of the Thai-Malay Peninsula Bent-toed Geckos of the *Cyrtodactyluspulchellus* complex (Squamata: Gekkonidae): combined morphological and molecular analyses with descriptions of seven new species. Zootaxa 3520: 1–55. 10.11646/zootaxa.3520.1.1

[B29] Grismer LL Wood Jr PL Anuar S Quah ESH Muin MA Chan KO Sumarli AX Loredo AI (2015) Repeated evolution of sympatric, palaeoendemic species in closely related, co-distributed lineages of *Hemiphyllodactylus* Bleeker, 1860 (Squamata: Gekkonidae) across a sky-island archipelago in Peninsular Malaysia. Zoological Journal of the Linnean Society 174(4): 859–876. 10.1111/zoj.12254

[B30] Grismer LL Wood Jr PL Anuar S Davis HR Cobos AJ Murdoch ML (2016) A new species of karst forest Bent-toed Gecko (genus *Cyrtodactylus* Gray) not yet threatened by foreign cement companies and a summary of Peninsular Malaysia’s endemic karst forest herpetofauna and the need for its conservation. Zootaxa 4061: 1–17. 10.11646/zootaxa.4061.1.1 27395475

[B31] Grismer LL Wood Jr PL Aowphol A Cota M Grismer MS Murdoch ML Aguilar C Grismer JL (2017) Out of Borneo, again and again: biogeography of the Stream Toad genus *Ansonia* Stoliczka (Anura: Bufonidae) and the discovery of the first limestone cave-dwelling species. Biological Journal of the Linnean Society 120: 371–395. 10.1111/bij.12886

[B32] Grismer LL Wood Jr PL Quah ESH Anuar S Ngadi EB Amalina Mohd Izam N Ahmad N (2018a) Systematics, ecomorphology, cryptic speciation and biogeography of the lizard genus *Tytthoscincus* Linkem, Diesmos & Brown (Squamata: Scincidae) from the sky- island archipelago of Peninsular Malaysia. Zoological Journal of the Linnean Society 183: 635–671. 10.1093/zoolinnean/zlx067

[B33] Grismer LL Rujirawan A Termprayoon K Ampai N Yodthong S Wood Jr PL Oaks JR Aowphol A (2020a) A new species of *Cyrtodactylus* Gray (Squamata; Gekkonidae) from the Thai Highlands with a discussion on the evolution of habitat preference. Zootaxa 4852: 401–427. 10.11646/zootaxa.4852.4.1 33056404

[B34] Grismer LL Wood Jr PL Quah ESH Grismer MS Myint Kyaw Thura Oaks JR Aung Lin (2020b) Two new species of *Cyrtodactylus* Gray, 1827 (Squamata: Gekkonidae) from a karstic archipelago in the Salween Basin of southern Myanmar (Burma). Zootaxa 4718: 151–183. 10.11646/zootaxa.4718.2.1 32230014

[B35] Grismer LL Chan KO Oaks JR Neang T Sokun L Murdoch ML Stuart BL Grismer JL (2020c) A new insular species of the *Cyrtodactylusintermedius* (Squamata: Gekkonidae) group from Cambodia with a discussion of habitat preference and ecomorphology. Zootaxa 4830: 75–102. 10.11646/zootaxa.4830.1.3 33056252

[B36] Grismer LL Geissler P Neang T Hartmann T Wagner P Poyarkov NA (2021a) Molecular phylogenetics, PCA, and MFA recover a new species of *Cyrtodactylus* (Squamata: Gekkonidae) from an isolated sandstone massif in northwestern Cambodia. Zootaxa 4949: 261–288. 10.11646/zootaxa.4949.2.3 33903343

[B37] Grismer LL Wood Jr PL Poyarkov NA Le MD Karunarathna S Chomdej S Suwannapoom C Qi S Liu S Che J Quah ESH Kraus F Oliver PM Riyanto A Pauwels OSG Grismer JL (2021b) Karstic landscapes are foci of species diversity in the world’s third-largest vertebrate genus *Cyrtodactylus* Gray, 1827 (Reptilia: Squamata; Gekkonidae). Diversity 13: 183. 10.3390/d13050183

[B38] Grismer LL Wood PL Poyarkov NA Le MD Kraus F Agarwal I Oliver PM Nguyen SN Nguyen TQ Karunarathna S Welton LJ Stuart BL Luu VQ Bauer AM O’Connell KA Quah ESH Chan KO Ziegler T Ngo H Nazarov RA Aowphol A Chomdej S Suwannapoom C Siler CD Anuar S Tri NV Grismer JL (2021c) Phylogenetic partitioning of the third-largest vertebrate genus in the world, *Cyrtodactylus* Gray, 1827 (Reptilia; Squamata; Gekkonidae) and its relevance to taxonomy and conservation. Vertebrate Zoology 71: 101–154. 10.3897/vertebrate-zoology.71.e59307

[B39] Grismer LL Anuar S Muin MA Ahmad N Quah ESH (2023b) Genetic and morphological concordance and discordance within the *Cyrtodactylusbrevipalmatus* group (Squamata: Gekkonidae). Zootaxa 5353: 265–275. 10.11646/zootaxa.5353.3.4 38220685

[B40] Grismer LL Pawangkhanant P Idiiatullina SS Trofimets AV Nazarov RA Suwannapoom C Poyarkov NA (2023c) A new species of *Cyrtodactylus* Gray, 1827 (Squamata: Gekkonidae) from the Thai-Malay Peninsula and the independent evolution of cave ecomorphology on opposite sides of the Gulf of Thailand. Zootaxa 5352: 109–136. 10.11646/zootaxa.5352.1.4 38221458

[B41] Grismer LL Rujirawan A Chomdej S Suwannapoom C Yodthong S Aksornneam A Aowphol A (2023a) A new species of the *Cyrtodactylusbrevipalmatus* group (Squamata, Gekkonidae) from the uplands of western Thailand. ZooKeys 1141: 93–118. 10.3897/zookeys.1141.97624 37234966 PMC10207280

[B42] Grismer LL Aowphol A Grismer JL Aksornneam A Quah ESH Murdoch ML Gregory JJ Nguyen E Kaatz A Bringsøe H Rujirawan A (2024a) A new species of the *Cyrtodactyluschauquangensis* group (Squamata, Gekkonidae) from the borderlands of extreme northern Thailand. ZooKeys 1203: 211–238. 10.3897/zookeys.1203.122758 38855793 PMC11161685

[B43] Grismer LL Sinovas P Quah ESH Th I S Chourn P Chhin S Hun S Cobos C Geissler P Ching C Murdoch ML (2025) A new species of lowland karst-dwelling Slender Gecko *Hemiphyllodactylus* Bleeker, 1860 (Squamata: Gekkonidae) from a karstic archipelago in western Cambodia. Zootaxa 5569: 253–281. 10.11646/zootaxa.5569.2.3 40173545

[B44] He K Gutiérrez EE Heming NM Koepfli K Wan T He S Jin W Liu SY Jiang XL (2019) Cryptic phylogeographic history sheds light on the generation of species diversity in sky-island mountains. Journal of Biogeography 46: 2232–2247. 10.1111/jbi.13664

[B45] Hillis DM (2019) Species delimitation in herpetology. Journal of Herpetology 53: 3–12. 10.1670/18-123

[B46] Hoang DT Chernomor O von Haeseler A Minh BQ Vinh LS (2018) UFBoot2: Improving the ultrafast bootstrap approximation. Molecular Biology and Evolution 35: 518–522. 10.1093/molbev/msx281 29077904 PMC5850222

[B47] Huelsenbeck JP Ronquist F Nielsen R Bollback JP (2001) Bayesian inference of phylogeny and its impact on evolutionary biology. Science 294: 2310–2314. 10.1126/science.1065889 11743192

[B48] Husson F Josse J Le S Mazet J (2017) FactoMine R: exploratory data analysis and data mining. R package, version 1.36. 10.32614/CRAN.package.FactoMineR

[B49] Jombart T (2021) An introduction to *adagenet* 2.1.5.

[B50] Kaatz A Grismer JL Grismer LL (2021) Convergent evolution of karst habitat preference and its ecomorphological correlation in three species of Bent-toed Geckos (*Cyrtodactylus*) from Peninsular Malaysia. Vertebrate Zoology 71: 367–386. 10.3897/vz.71.e66871

[B51] Kalyaanamoorthy S Minh BQ Wong TK von Haeseler A Jermiin LS (2017) ModelFinder: fast model selection for accurate phylogenetic estimates. Nature Methods 14: 587. 10.1038/nmeth.4285 PMC5453245 28481363

[B52] Kassambara A Mundt F (2017) Factoextra: extract and visualize the result of multivariate data analyses. R package, version 1.0.5.999. 10.32614/CRAN.package.factoextra

[B53] Lleonart J Salat J Torres GJ (2000) Removing allometric effects of body size in morphological analysis. Journal of Theoretical Biology 205: 85–93. 10.1006/jtbi.2000.2043 10860702

[B54] Loredo AI Wood Jr PL Quah ESH Anuar S Greer LF Ahmad N Grismer LL (2013) Cryptic speciation within *Asthenodipsasvertebralis* (Boulenger, 1900) (Squamata: Pareatidae), the description of a new species from Peninsular Malaysia, and the resurrection of *A.tropidonotus* (Lidth de Jude, 1923) from Sumatra: an integrative taxonomic approach. Zootaxa 3664: 505–524. 10.11646/zootaxa.3664.4.5 26266316

[B55] Losos J (2009) Lizards in an Evolutionary Tree. University of California Press, Berkeley. 10.1525/9780520943735

[B56] Luo Z Tang S Jiang Z Chen J Fang H Li C (2016) Conservation of terrestrial vertebrates in a global hotspot of karst in southwestern China. Scientific Reports. 10.1038/srep25717 PMC4881395 27228463

[B57] Maddison WP Maddison DR (2015) Mesquite: a modular system for evolutionary analysis. Version 3.04. 10.1093/sysbio/42.2.218

[B58] Miller MA Pfeiffe W Schwartz T (2010) Creating the CIPRES Science Gateway for inference of large phylogenetic trees. In: Gateway Computing Environments Workshop (GCE), New Orleans (USA), November 2010, IEEE, 1–8. 10.1109/GCE.2010.5676129

[B59] Minh Q Nguyen MAT von Haeseler A (2013) Ultrafast approximation for phylogenetic bootstrap. Molecular Biology and Evolution 30: 1188–1195. 10.1093/molbev/mst024 23418397 PMC3670741

[B60] Murdoch ML Grismer LL Wood Jr. PL Neang T Poyarkov NA Ngo VT Nazarov RA Aowphol A Pauwels OSG Nguyen HN Grismer JL (2019) Six new species of the *Cyrtodactylusintermedius* complex (Squamata: Gekkonidae) from the Cardamom Mountains and associated highlands of Southeast Asia. Zootaxa 4554: 1–62. 10.11646/zootaxa.4554.1.1 30790979

[B61] Naomi S-I (2011) On the integrated frameworks of species concepts: Mayden’s hierarchy of species concepts and de Queiroz’s unified concept of species. Journal of Zoological Systematics and Evolutionary Research 49: 177–184. 10.1111/j.1439-0469.2011.00618.x

[B62] Nguyen LT Schmidt HA von Haeseler A Minh BQ (2015) IQ-TREE: A fast and effective stochastic algorithm for estimating maximum likelihood phylogenies. Molecular Biology and Evolution 32: 268–274. 10.1093/molbev/msu300 25371430 PMC4271533

[B63] Oksanen J Blanchet FG Friendly M Kindt R Legendre P McGlinn D Minchin PR O’Hara RB Simpson GL Solymos P Stevens MHH Szoecs E Wagner H (2020) Package ‘vegan’. Version 2.5–7. https://cran.r-project.org/web/packages/vegan/

[B64] Padial JM Miralles A de la Rivera I Vences M (2010) The integrative future of taxonomy. Frontiers in Zoology 7: 1–14. 10.1186/1742-9994-7-16 20500846 PMC2890416

[B65] Pagès J (2015) Multiple Factor Analysis by Example Using R. CRC Press, New York, 272 pp. 10.1201/b17700-10

[B66] Pauwels OSG Chotjuckdikul N Donbundit N Sumontha M Meesook W (2024) *Cyrtodactyluspanitvongi*, a new cave-dwelling Bent-toed Gecko from Lopburi Province, central Thailand (Squamata: Gekkonidae). Zootaxa 5512: 373–388. 10.11646/zootaxa.5512.3.3 39647055

[B67] Quah ESH Anuar S Grismer LL Wood Jr PL Azizah Mohd Nor S (2019) Systematics and natural history of mountain reed snakes (genus *Macrocalamus*; Calamariinae). Zoological Journal of the Linnean Society 188: 1236–1276. 10.1093/zoolinnean/zlz092

[B68] Quah ESH Grismer LL Shahrul AMS (2021) Conservation of Peninsular Malaysia’s Karst Herpetofauna: A review of herpetological discoveries, research trends, and challenges. Raffles Bulletin of Zoology 69: 235–252.

[B69] R Core Team (2020) R: A language and environment for statistical computing. R foundation for statistical computing, Vienna, Austria. https://www.R-project.org

[B70] Rambaut A Drummond AJ (2013) TreeAnnotator v1.8.0 MCMC Output Analysis. 10.1017/CBO9780511819049.020

[B71] Ramírez-Reyes T Blair C Flores-Villela O Pieroe D Lathropf A Murphy R (2020) Phylogenomics and molecular species delimitation reveals great cryptic diversity of leaf-toed geckos (Phyllodactylidae: *Phyllodactylus*), ancient origins, and diversification in Mexico. Molecular Phylogenetics and Evolution 150: 106880. 10.1016/j.ympev.2020.106880 32512192

[B72] Reilly SB Stubbs AL Karin BR Arida E Arifin U Hamidy A Kaiser H Bi K Riyanto A Iskandar DT McGuire JA (2023) Bewildering biogeography: Waves of dispersal and diversification across southern Wallacea by bent-toed geckos (genus: *Cyrtodactylus*). Molecular Phylogenetics and Evolution 186: 1–14. 10.1016/j.ympev.2023.107853 37327831

[B73] Reist JD (1986) An empirical evaluation of coefficients used in residual and allometric adjustment of size covariation. Canadian Journal of Zoology 64: 1363–1368. 10.1139/z86-203

[B74] Ronquist F Teslenko M Van Der Mark P Ayres DL Darling A Höhna S Larget B Liu L Suchard MA Huelsenbeck JP (2012) MrBayes 3.2: Efficient Bayesian phylogenetic inference and model choice across a large model space. Systematic Biology 61(3): 539–542. 10.1093/sysbio/sys029 22357727 PMC3329765

[B75] Skalski JR Richins SM Townsend RL (2018) A statistical test and sample size recommendations for comparing community composition following PCA. PLOS ONE 13: e0206033. 10.1371/journal.pone.0206033 PMC6200243 30356253

[B76] Sodhi NS Brook BW (2006) Southeast Asian Biodiversity in Crisis, Cambridge University Press. 10.1093/auk/123.1.275

[B77] Strijk JS Noyes RD Strasberg D Cruaud C Gavory F Chase MW Abbott RJ Thébaud C (2012) In and out of Madagascar: dispersal to peripheral islands, insular speciation and diversification of Indian Ocean Daisy Trees (*Psiadia*, Asteraceae). PLoS ONE 7(8): e42932. 10.1371/journal.pone.0042932 PMC3416790 22900068

[B78] Sumontha M Panitvong N Kunya K Donbundit N Suthanthanhjai W Suthanthangjai M Phanampho NE Pauwels OSG (2024) Two new cave-dwelling species of Bent-toed Geckos from Saraburi Loei provinces, Thailand (Squamata: Gekkonidae: *Cyrtodactylus*). Zootaxa 5512: 272–294. 10.11646/zootaxa.5512.2.9 39647059

[B79] Swann DE Mau-Crimmins TM Stitt EW (2005) In search of the Madrean line: biogeography of herpetofauna in the sky island region. In: Gottfried GJ Gebow BS Eskew LG Edminster CB (Eds) Connecting mountain islands and desert seas: biodiversity and management of the Madrean Archipelago II.USDA Forest Service RMRS-P-36, 149–153.

[B80] Tamura K Stecher G Kumar S (2021) MEGA11: Molecular evolutionary genetics analysis version 11. Molecular Biology and Evolution 38: 3022–3027. 10.1093/molbev/msab120 33892491 PMC8233496

[B81] Termprayoon K Rujirawan A Grismer LL Wood Jr PL Aowphol A (2023) ﻿Two new karst-adapted species in the *Cyrtodactyluspulchellus* group (Reptilia, Gekkonidae) from southern Thailand. ZooKeys 1179: 313–352. 10.3897/zookeys.1179.109712 37745621 PMC10514696

[B82] Thorpe RS (1975) Quantitative handling of characters useful in snake systematics with particular reference to intraspecific variation in the Ringed Snake *Natrixnatrix* (L.). Biological Journal of the Linnean Society 7: 27–43. 10.1111/j.1095-8312.1975.tb00732.x

[B83] Thorpe RS (1983) A review of the numerical methods for recognising and analysing racial differentiation. In: Felsenstein J (Ed.) Numerical Taxonomy.NATO ASI Series, Volume 1. Springer, Berlin, Heidelberg, 404–423. 10.1007/978-3-642-69024-2_43

[B84] Trifinopoulos J Nguyen LT von Haeseler A Minh BQ (2016) W-IQ-TREE: a fast online phylogenetic tool for maximum likelihood analysis. Nucleic Acids Research 44: W232–235. 10.1093/nar/gkw256 PMC4987875 27084950

[B85] Turan C (1999) A note on the examination of morphometric differentiation among fish populations: The Truss System. Turkish Journal of Zoology 23: 259–263.

[B86] Wallace AR (1869) The Malay Archipelago. Macmillan & Co., London. 10.2307/3025162

[B87] Wang K Zhang C Chen H Yue Y Zhang W Zhang M Qi X Fu Z (2019) Karst landscapes of China: patterns, ecosystem processes and services. Landscape Ecology 34: 2743–2763. 10.1007/s10980-019-00912-w

[B88] Wiens JJ Camacho A Goldberg A Jezkova T Kaplan ME Lambert SM Miller EC (2019) Climate change, extinction, and sky island biogeography in a montane lizard. Molecular Ecology 28: 2610–24. 10.1111/mec.15073 30843297

[B89] Wilcox T Zwickl DJ Heath TA Hillis DM (2002) Phylogenetic relationships of the Dwarf Boas and a comparison of Bayesian and bootstrap measures of phylogenetic support. Molecular Phylogenetics and Evolution 25: 361–371. 10.1016/S1055-7903(02)00244-0 12414316

[B90] Williams EE (1972) The origin of faunas. Evolution of lizard congeners in a complex island fauna: a trial analysis. Evolutionary Biology 6: 47–89. 10.1007/978-1-4684-9063-3_3

[B91] Wood Jr PL Grismer LL Muin MA Anuar S Oaks JR (2020) A new potentially endangered limestone-associated Bent-toed Gecko of the *Cyrtodactyluspulchellus* (Squamata: Gekkonidae) complex from northern Peninsular Malaysia. Zootaxa 4751: 437–460. 10.11646/zootaxa.4751.3.2 32230404

